# Distinct Swimming Behaviours in Pupae of *Aedes*, *Anopheles* and *Culex* Mosquitoes

**DOI:** 10.1002/ece3.74061

**Published:** 2026-07-26

**Authors:** Christian Drerup, Mingyue Feng, Jet S. Griep, Kyle J. Walker, Marta Villa, Amir Atapour‐Abarghouei, Sara Epis, Marcus S. C. Blagrove, Olena Riabinina

**Affiliations:** ^1^ Department of Biosciences Durham University Durham UK; ^2^ Department of Entomology, College of Plant Protection China Agricultural University Beijing China; ^3^ Institute of Infection, Veterinary and Ecological Sciences University of Liverpool Liverpool UK; ^4^ Department of Biosciences University of Milan Milan Italy; ^5^ Department of Computer Science Durham University Durham UK

**Keywords:** adaptation, disease vector, ecology, habitat

## Abstract

Insect pupae are commonly considered an immobile, ecologically inactive life stage. Mosquito pupae, however, differ strongly from other holometabolous pupae in their mobility levels as they can actively move through their aquatic habitat. Despite this largely unique behavioural feature amongst insects, little is known about the pupal swimming behaviour and its associated costs and benefits in mosquitoes. As an improvement of our understanding of the ecology of mosquitoes, in particular of their juvenile stages, is required to develop or refine control strategies to prevent the transmission of deadly diseases, we here investigated the pupal surface swimming behaviour in 11 mosquito species of the genera *Aedes*, *Anopheles* and *Culex*. We observed distinct differences in their swimming behaviour, with *Aedes* pupae generally characterised by slow yet frequent movement bouts and high explorative behaviour, whereas *Anopheles* and especially *Culex* exhibit significantly fewer movement bouts but swim faster and cover larger distances per movement bout. These behavioural differences may reflect ecological adaptations tailored to the specific habitats of the tested species. In summary, our findings reveal a previously undescribed behavioural dissimilarity amongst species in this frequently overlooked mosquito juvenile life stage and highlight the importance of considering pupal behaviour when developing and improving mosquito disease control strategies.

## Graphical abstract

As improving our understanding of the ecology of mosquitoes, in particular of their juvenile stages, is required to develop or refine control strategies to prevent the transmission of deadly diseases, we here investigated the pupal surface swimming behaviour in 11 mosquito species of the genera *Aedes*, *Anopheles* and *Culex*. We observed distinct differences in their swimming behaviour, with *Aedes* pupae generally characterised by slow yet frequent movement bouts and high explorative behaviour, whereas Anopheles and especially *Culex* pupae exhibit significantly fewer movement bouts but swim faster and cover larger distances per movement bout. Our findings reveal a previously undescribed behavioural dissimilarity amongst species in this frequently overlooked mosquito juvenile life stage and may reflect ecological adaptations tailored to the specific habitats of the tested species.

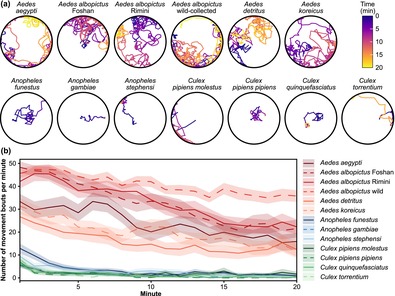

## 1 Introduction

Mosquitoes (Diptera: Culicidae) are global vectors of many infectious diseases such as malaria, dengue, Zika, chikungunya and West Nile fever (Weaver et al. 2018). Although numerous control strategies, ranging from insecticide‐based interventions and habitat modification to biological and genetic technologies, currently exist (Wilson et al. 2020; Godfray 2013), their effectiveness ultimately relies on understanding the ecological and behavioural traits of mosquito populations (Chandrasegaran, Lahondère, et al. 2020). In particular, knowledge of mosquito ecology allows us to determine when and where transmission occurs, which interventions are effective, how mosquitoes respond or adapt to control pressures and how populations change across landscapes and seasons (LaDeau et al. 2015; Egid et al. 2022).

The mosquito life cycle consists of aquatic egg, larval and pupal stages, from which the terrestrial, flying adults eclose (Hawkes and Hopkins 2021). While ecological aspects of the egg, larval and adult phases are frequently investigated, the pupal stage has received far less attention despite its importance in the final development of the adults (Rolff et al. 2019). As for all species of holometabolous insects, the pupal stage is considered a developmental phase in which larval tissue and organs are remodelled into their adult counterparts (Truman 2019; Hall and Martín‐Vega 2019). As such, it is considered an ecologically inactive life phase throughout which pupae do not feed and remain immobile (Lindstedt et al. 2019). Mosquito pupae, however, defy this norm and differ strongly from other holometabolous pupae in their activity and mobility levels, as they can use their abdomens to actively move through their aquatic habitat (Brackenbury 1999).

Mosquito pupae are positively buoyant and commonly rest at the water surface (Romoser 1975). In response to physical or visual disturbance, however, they can dive into the water column, thereby avoiding predatory attacks (Rodríguez‐Prieto et al. 2006; Awasthi et al. 2012; Futami et al. 2008; Chandrasegaran, Sriramamurthy, et al. 2020; Cordeschi et al. 2025) or being flushed out of their aquatic environment (Koenraadt and Harrington 2008). Pupae can also swim along the water surface (Brackenbury 1999); however, the costs and benefits of this surface swimming behaviour have not yet been identified. Surface swimming might potentially allow mosquito pupae to interact with their environment, for example through avoiding habitats with strong predation risks, or reaching habitats better suited for successful eclosion. However, considering that food intake ceases at pupal stage (Ratnayake et al. 2023), active movement at this life stage strongly contributes to a depletion of the energy reserves obtained from the larval stage, which in turn might interfere with metamorphosis and successful adult eclosion and survival (Lucas and Romoser 2001). Therefore, mosquito pupae must balance their need for active movement with the corresponding expenditure of the limited energy reserves.

A recent investigation of the swimming behaviour of mosquito larvae revealed distinct locomotion patterns and strong variations in the activity, speed and distance travelled between species (Lutz et al. 2020). While it has been hypothesised that both phylogenetic relatedness and ecological specialisation have caused these behavioural differences at the larval stage (Lutz et al. 2020), it remains unknown whether similar species‐specific differences also exist in swimming behaviour and activity of mosquito pupae. The presence of such differences, however, might indicate ecological adaptations shaped by a trade‐off between movement‐related benefits and the limited energy reserves in this non‐feeding life stage.

Here, we investigate the surface swimming behaviour of mosquito pupae from 11 species across the genera *Aedes*, *Anopheles* and *Culex*, which comprise the majority of mosquito disease vectors worldwide (Wei et al. 2026). By quantifying behavioural parameters under controlled laboratory conditions, we provide new insights into the ecological diversity of pupal behaviour across mosquito genera and species, and whether these differences can be explained by phylogenetic relatedness or specific ecological requirements. Our study, therefore, aims to improve our understanding of the behavioural ecology of this often‐overlooked mosquito juvenile life stage, which might further help with the development of control strategies specifically targeted to the pupal phase of mosquitoes.

## 2 Material and Methods

### 2.1 Mosquito Rearing

A total of 11 species were used in this study: *Aedes aegypti* , *Aedes albopictus* (three different strains), *Aedes detritus*, *Aedes koreicus*, *Anopheles funestus*, *Anopheles gambiae* , *Anopheles stephensi*, *Culex pipiens* bioform *molestus*, *Culex pipiens* bioform *pipiens*, *Culex quinquefasciatus* and *Culex torrentium*. While for most species pupae were derived from established laboratory strains (Table 1), we also tested pupae from wild populations collected specifically for this project. In particular, larvae of *Ae. albopictus* (identified based on morphological traits (Becker et al. 2020)) were collected in suburban Milan (Northern Italy; 45°25′18.9″N 9°15′48.5″ E) and brought to the campus of the University of Milan where they were reared to the pupal stage. Similarly, larvae of *Ae. detritus* (identified based on molecular methodologies presented below) were collected in Little Neston (North‐East England; 53°16′37.2″ N, 3°04′06.4″ W) and brought to the University of Durham where they were reared to the pupal stage. Lastly, egg rafts of *Culex* sp. were collected from and reared to larval stage at the University of Liverpool's Leahurst Campus (North‐East England; 53°17′24.2″N 3°01′37.5″W) before being transported to the University of Durham where they were reared to pupal stage. All tested pupae derived from these egg rafts were identified based on methodologies presented below, revealing individuals from both *Cx. p. pipiens* and *Cx. torrentium*. For all tested species, larvae and pupae were reared in shallow plastic trays under a photoperiodic cycle of 12:12 h (light:dark) and fed *ad libitum* with either fish food (either Staple Food Pellets, Nishikoi Aquaculture Ltd.; or Goldfish Gold Energy, Tetra Werke, Germany) or yeast tablets (Brewer's Yeast tablets, Holland & Barrett Retail Limited, UK). For all species maintained as colonies, adults were kept in mixed‐sex cages and fed with 10% sucrose solution.

**TABLE 1:** Species used in the present study.

Species	Strain	Time in captivity	*N* total	*N* ♂	*N* ♀	Institute	Temp.	RH
*Aedes aegypti*	Liverpool	90 years (since 1936)	26	12	14	Liverpool	27°C	70%
*Aedes albopictus*	Foshan	44 years (since 1981)	26	11	15	Milan	27°C	70%
*Aedes albopictus*	Rimini	21 years (since 2004)	26	10	16	Milan	27°C	70%
*Aedes albopictus*	Wild‐collected	n/a	26	14	12	Milan	27°C	70%
*Aedes detritus*	Wild‐collected	n/a	27	13	14	Durham	20°C	50%
*Aedes koreicus*	Como	4 years (since 2021)	26	13	13	Milan	25°C	60%
*Anopheles funestus*	FuMoz	25 years (since 2000)	30	17	13	Durham	27°C	70%
*Anopheles gambiae*	G3	50 years (since 1975)	34	16	18	Durham	27°C	70%
*Anopheles stephensi*	SDA500	43 years (since 1982)	26	9	17	Milan	27°C	70%
*Culex pipiens molestus*	Mogden	3 years (since 2022)	33	12	21	Leahurst	25°C	60%
*Culex pipiens pipiens*	Wild‐collected	n/a	17	9	8	Durham	20°C	50%
*Culex quinquefasciatus*	Muheza	40 years (since 1985)	34	12	22	Durham	27°C	70%
*Culex torrentium*	Wild‐collected	n/a	17	5	12	Durham	20°C	50%

*Note:* Institute = Research institute where the behavioural experiment was conducted Durham = Department of Biosciences, University of Durham, Durham, UK; Leahurst = Leahurst Campus, School of Veterinary Science, University of Liverpool, Leahurst, UK; Liverpool = Biosciences Building, University of Liverpool, Liverpool, UK; Milan = Department of Biosciences, University of Milan, Milan, Italy.

Abbreviations: *N*, number of tested individuals; RH, rearing relative humidity; Temp., rearing temperature.

### 2.2 Molecular Identification of Field‐Collected Mosquitoes

DNA was extracted from the whole body (DNeasy Blood and Tissue Kit; Qiagen, USA), following the manufacturer's protocol with minor modifications: the mosquito bodies were homogenised in 180 μL of buffer ATL, followed by the addition of 20 μL of proteinase K and incubation at 56°C for at least 30 min. Manufacturer's instructions were followed from this point onwards. At the final elution step, we eluted the DNA with 50 μL of molecular grade water. The cytochrome c oxidase subunit I (COI) gene was amplified using the universal primers LCO1490/HCO2198 and C1‐J‐2183/TL2‐N‐3014 (Pat) (Folmer et al. 1994; Simon et al. 1994). PCR products were verified by agarose gel electrophoresis and subsequently sequenced by GENEWIZ (Azenta Life Sciences) using Sanger sequencing. Sequences were then aligned against the National Centre for Biotechnology Information (NCBI) database using Basic Local Alignment Search Tool, Nucleotide version (BLASTn).

Samples identified as *Culex* spp. were further examined using diagnostic restriction enzyme digestion of the COI region with FspBI (BfaI) and SspI (FastDigest FspBI and FastDigest SspBI; Thermo Fisher Scientific Inc., Waltham, MA, USA) following established protocols (Hesson et al. 2010) to distinguish *Cx. torrentium* from the *Cx. pipiens* complex. Remaining individuals within the *Cx. pipiens* complex were identified using PCR with primers molCQ11R/pipCQ11R and CQ11F2 (Bahnck and Fonseca 2006) to differentiate between *Cx. p*. *pipiens* and *Cx. p. molestus*. For all subsequent analysis, we included only data from wild‐caught individuals that were clearly identified as *Ae. detritus*, *Cx. p. pipiens*, or *Cx. torrentium* (in addition to the wild‐collected *Ae. albopictus* individuals identified based on morphological rather than molecular traits).

### 2.3 Experimental Setup and Procedure

Assessment of pupal swimming behaviour took place in a circular arena consisting of a white silicone mould (290 mm diameter, 13 mm deep) filled with 400 mL of water taken from each species' rearing tray, resulting in a water level of approximately 6 mm. This shallow water depth was intentionally selected to constrain vertical movement and enable standardised quantification of surface swimming behaviour across all mosquito species. The arena was placed at the bottom of a cubical, 400 × 400 × 400 mm wide photography box (DUCLUS Light Box 40 cm; hereinafter simplified as box), which offered a small opening at the top for top‐down directed video recordings and a larger, sealable side opening for pupae handling. A camera (α7S III with FE 24–105 mm f4 G OSS lens, Sony Group Corporation, Tokyo, Japan) was mounted above the box so that the lens set flush with the top opening of the box. The camera was connected to an external monitor via its HDMI port, allowing observation of pupae activity remotely throughout trials. Lighting was provided by the box's inbuilt LED ring consisting of 160 LED beads with a colour temperature of 5500 K. A diffusor cloth, with a centrally cut opening to not obstruct video recordings, was hung below the LED ring to disperse light equally across the box, resulting in a light intensity of 840 lx at the bottom of the box. As mosquito pupae were tested across different research institutes (Table 1), the experimental setup (photography box with top‐mounted camera) was either placed within large incubators or on workbenches in climate‐controlled rooms to match each species' rearing conditions. Using the same experimental setup for each species, we ensured that across all tested pupae the lighting levels, visual scene and experimental procedures were identical. The water temperature of the experimental arena was consistent with each species' rearing conditions, resulting in temperature conditions ranging from 20°C for temperate species to 27°C for tropical species (Table 1). To assess whether these temperature differences may explain behavioural differences amongst the tested species, we additionally recorded pupae of one representative temperate species (*Ae. detritus*; *N* = 25; ♂ = 8; ♀ = 17) at 27°C and of one representative tropical species (*An. gambiae*; *N* = 27; ♂ = 12; ♀ = 15) at 20°C water temperature.

To run trials, groups of up to five pupae were collected from their rearing trays and collectively transferred into a small container filled with rearing water. This container was placed into a corner of the photography box to allow pupae to acclimatise to the experimental lighting levels. After 10 min of acclimatising, one pupa was individually collected from this container using a Pasteur pipette and carefully released in the centre of the arena. After closing the door of the photography box, the pupal swimming behaviour was recorded using the top‐mounted camera for 20 min (30 fps; 1920 × 1080 px). After each trial, the pupa was collected from the arena with a Pasteur pipette and its sex was determined by assessing sex‐specific morphological differences in either the pupal genital lobe shape under the microscope (all *Aedes* and *Anopheles* species (Moorefield 1951)) or the antennae of adults reared from pupal stage (all *Culex* species (Bansal and Sen 2025)). To avoid age‐specific variation in pupal behaviour, all pupae were tested at an intermediate stage of the pupal period, thereby excluding both recently pupated individuals and those close to eclosion. All trials across all species were conducted during daytime (09:00–18:00 h) by the same experimenter.

### 2.4 Trajectory Analysis

All video recordings were analysed using idtracker.ai (https://idtracker.ai/) (Romero‐Ferrero et al. 2019; Torrents et al. 2026). Using the same settings for all individuals across all species (background subtraction enabled and set to ‘max’, background difference threshold = 40, blob area thresholds between 1 and 100), we established the x‐y coordinates and corresponding trajectory path of each pupa across all frames of a video recording. The accuracy of each trajectory was assessed through visual inspection as well as by identifying the proportion of frames a pupa was tracked throughout a trial, with each pupa being tracked in at least 99.31% of the 36,000 frames of its trial. The x‐y coordinates obtained from idtracker.ai were then imported into MATLAB (The Mathworks Inc.; Natick, MA, U.S.A.) where each pupal trajectory was smoothed using MATLAB's *smooth* function (Savitzky–Golay method; span = 5; degree = 1). We then calculated the instantaneous speed of pupae as the distance travelled between two consecutive frames, followed by the conversion of each distance from pixel width to mm by using the real‐world dimension of the arena as a size reference. Log‐transforming these distances revealed a bimodal distribution, of which the trough between both peaks defined the threshold (0.21 mm per frame) for stationary (speed below threshold) or moving behaviour (speed above threshold).

**FIGURE A1:**
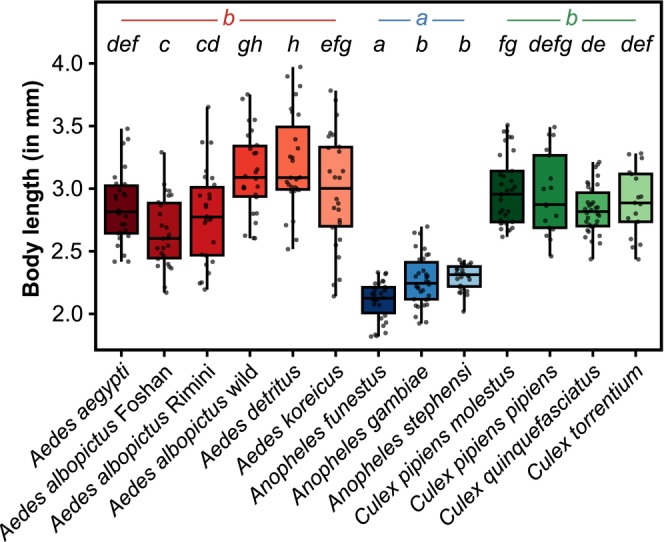
Pupal body length across all tested species. For each species, a boxplot visualises the body length distribution, whereas jittered grey circles represent the raw data for each individual. All boxplots represent the median (horizontal line) and quartiles (upper and lower boundaries) of a species' distribution, with the whiskers extending to the most extreme measurement within 1.5 times the interquartile range. Letters represent statistically similar groups based on statistical models assessing body length as a function of either genus or species and corresponding post hoc analysis (*p* < 0.05), with the first row of coloured letters depicting statistically similar groups amongst the three genera *Aedes* (red), *Anopheles* (blue) and *Culex* (green), whereas the second row comprising black letters depicts statistically similar groups amongst all 13 tested species/strains.

We calculated several behavioural metrics to establish potential differences in pupal swimming activity. Movement latency was calculated as the time passed until the pupal speed first exceeded the movement threshold. Number of movement bouts was calculated as continuous (> 3) series of speeds above the movement threshold separated by speeds below the threshold. Initial activity was calculated as the sum of all movement bouts (as defined above) that occurred within the first 10% (2 min) of the trial. Time spent stationary was calculated as the total instances in which the speed of an individual was below the movement threshold. Total swimming distance was calculated as the sum of all distances travelled for instances in which a pupa was moving. Arena exploration, defined as the proportion of area within the arena that a pupa explored, was calculated by placing a 5 mm wide square grid over the arena, resulting in a total of 2644 squares covering the arena. Using this segmentation in combination with our obtained x‐y coordinates, we then calculated the proportional arena exploration as the sum of all squares visited by a pupa divided by the total number of squares. In addition to these swimming activity metrics, we also calculated two behavioural metrics describing the swimming performance: swimming speed and average displacement per movement bout. To normalise for size‐specific differences amongst the tested species (Figure A1), we converted these behavioural metrics into body length. The body length (BL) of each pupa was measured as the distance between the central point between the trumpets and the posterior end of the third abdominal segment in three randomly selected frames of its video recording, averaged and converted from pixels to mm using the same conversion rate as used for the pupae trajectories. We then calculated for each individual the average speed (in BL s^−1^) as the median of all speeds above the movement threshold divided by its body length. We used the median instead of the mean because it is less sensitive to occasional tracking artefacts in frame‐by‐frame speed estimates. Lastly, we established for each individual the average displacement (in BL) as the mean distance travelled per movement bout divided by an individual's body length.

### 2.5 Statistical Analysis

All statistical analyses were performed in R v. 4.4.3 (R Core Team 2025). Statistical models were fitted using the *lme4* (Bates et al. 2015), *glmmTMB* (Brooks et al. 2017) and *MASS* (Venables and Ripley 2002) packages, and model assumptions were assessed using the *performance* (Lüdecke et al. 2021) and *DHARMa* (Hartig 2022) packages. Where statistical approaches involved multiple pairwise comparisons, *p*‐values were adjusted using the Benjamini‐Hochberg method (Benjamini and Hochberg 1995). Pairwise differences amongst levels of fixed effects were analysed using the *emmeans* package (Lenth 2022), with statistically similar fixed effects grouped using the *multcomp* package (Hothorn et al. 2008). All data visualisations were rendered using the *ggplot2* package (Wickham 2016).

We first investigated whether the behaviours we analysed differed between species. This analysis included only observations from pupae exposed to their regular rearing water temperatures (Table 1), thus excluding observations from the two temperature control experiments (*Ae. detritus* at 27°C; *An. gambiae* at 20°C). For each of the eight behavioural metrics (movement latency, initial activity, time spent stationary, number of movement bouts, total distance, arena exploration, average speed and average displacement), we fitted two (generalised) linear models with the respective behavioural metric as the response variable and either species or genus as a categorical fixed effect. Specifically, we fitted linear models (LMs) for the movement latency (log‐transformed), initial activity, number of movement bouts, total distance (all square root‐transformed) and average speed (log‐transformed). In addition, we fitted generalised linear models (GLM) with appropriate error distributions for the time spent stationary (beta distribution), arena exploration (beta‐binomial distribution) and the average displacement (gamma distribution). For each behavioural metric, species and genus were analysed in two separate models owing to species being fully nested within genus, leading to perfect collinearity when included simultaneously. We further analysed whether pupal activity differed over time between species by fitting a GLM with a Poisson distribution, which included the number of movement bouts per minute as the response variable, an interaction term between minute and species as fixed effects and individual ID as a random effect.

We then investigated whether observed similarities and differences between species could be explained through phylogenetic relationships. To do this, we pooled all behavioural observations together and standardised them to zero mean and unit variance, including only individuals with complete data across all variables in the subsequent analysis. We then calculated a Euclidian distance matrix from the standardised behavioural variables using the ‘vegdist’ function and performed two permutational multivariate analyses of variation (perMANOVAs) using the ‘adonis2’ function (both *vegan* package (Oksanen et al. 2025)) with 9999 permutations to test for differences at both species and genus level. Subsequently, we performed a principal component analysis (PCA) based on our standardised behavioural variables using the prcomp function. Only PC1 and PC2 explained a significant proportion of the data variance (permutation test: PC1: 63.11%, *p* < 0.001; PC2: 20.25%, *p* < 0.001; all remaining PCs: *p* > 0.05). To assess how well genus explains the behavioural variation captured by the first two PCs, we fitted linear models for PC1 and PC2, and a MANOVA for the combined PC1‐PC2 space, with genus as a categorical fixed effect. To further explore patterns of similarity between species, we computed pairwise Euclidean distances and performed pairwise perMANOVAs by creating reduced datasets containing only observations for a given species pair. Obtained effect sizes (*R* ^*2*^) were visualised as a similarity heatmap. Lastly, we assessed hierarchical relationships between species in terms of their swimming behaviour by performing clustering using the ‘hclust’ function and Ward's method (Ward.D2) on the obtained Euclidean distance matrix.

To test whether swimming behaviour within a species is correlated with sex or body length, we created a reduced dataset for each species containing only individuals recorded at their standard rearing temperature. For each of the eight behavioural metrics, we then fitted (generalised) linear models with the respective behavioural variables as the response variable, sex as a categorical fixed effect and size as a continuous fixed effect, using the same model types as described for the species comparison above. To establish whether the swimming behaviour of mosquito pupae might be influenced by water temperature, we analysed a reduced data set comprising only observations from both *Ae. detritus* and *An. gambiae* at both 20°C and 27°C. For each of the eight tested behaviours, we fitted (generalised) linear models with the respective behavioural metric as the response variable and the interaction between species and temperature as categorical fixed effects, using the same model types as described for the species comparison above.

## 3 Results

### 3.1 Species Comparison

**FIGURE 1:**
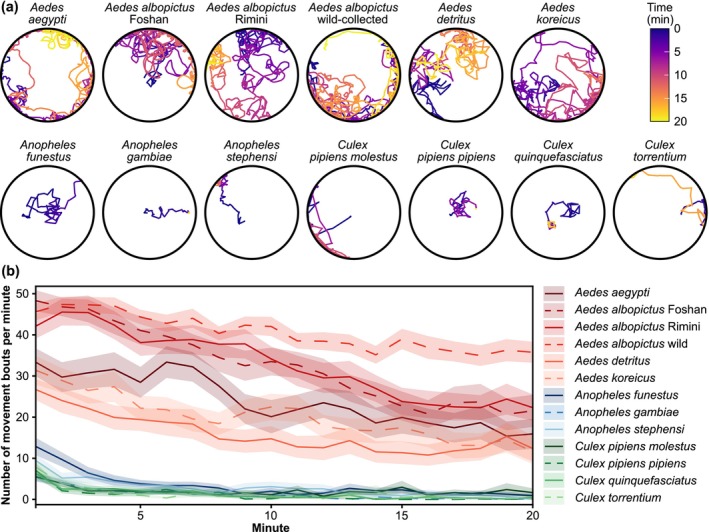
Trajectory paths and activity of mosquito pupae throughout the 20‐min‐long observation period. (a) Exemplary trajectories of the swimming activity of the 13 tested mosquito species. For each species, the individual closest to the species' mean swimming distance is shown. (b) Averaged (mean ± SE) number of movement bouts per minute for each species, including *Ae. aegypti* (*N* = 26; ♂ = 12; ♀ = 14), *Ae. albopictus* Foshan (*N* = 26; ♂ = 11; ♀ = 15), *Ae. albopictus* Rimini (*N* = 26; ♂ = 10; ♀ = 16), *Ae. albopictus* wild (*N* = 26; ♂ = 14; ♀ = 12), *Ae. detritus* (*N* = 27; ♂ = 13; ♀ = 14), *Ae. koreicus* (*N* = 26; ♂ = 13; ♀ = 13), *An. funestus* (*N* = 30; ♂ = 17; ♀ = 13), *An. gambiae* (*N* = 34; ♂ = 16; ♀ = 18), *An. stephensi* (*N* = 26; ♂ = 9; ♀ = 17), *Cx. p*. *molestus* (*N* = 33; ♂ = 12; ♀ = 21), *Cx. p*. *pipiens* (*N* = 17; ♂ = 9; ♀ = 8), *Cx. quinquefasciatus* (*N* = 34; ♂ = 12; ♀ = 22) and *Cx. torrentium* (*N* = 17; ♂ = 5; ♀ = 14).

**FIGURE 2:**
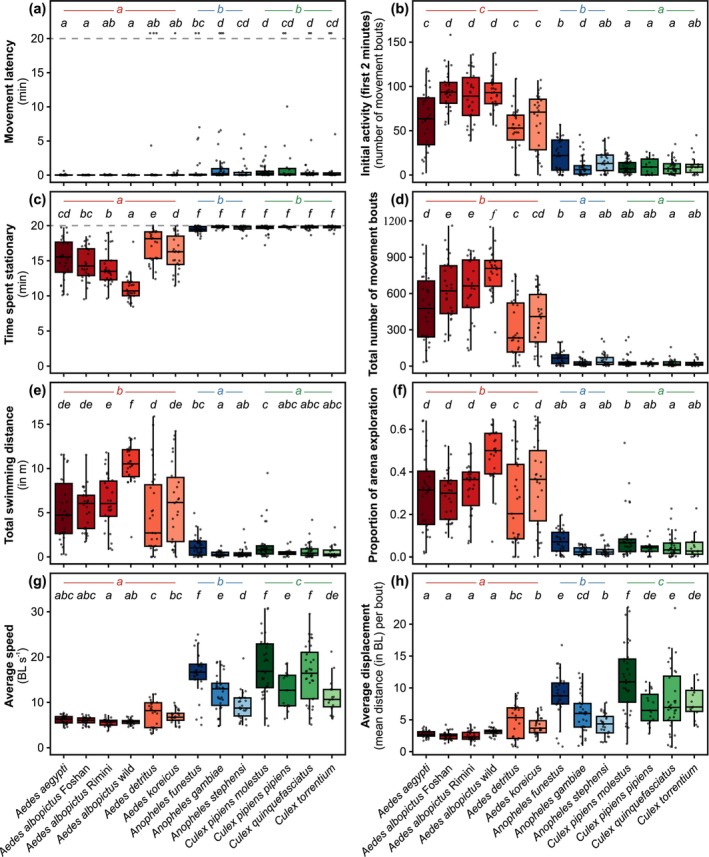
Behavioural variation in the swimming activity of mosquito pupae. For each quantified behaviour, boxplots represent the median (horizontal line) and quartiles (upper and lower boundaries) of a species' distribution, with the whiskers extending to the most extreme measurement within 1.5 times the interquartile range, and jittered grey points representing individual raw data. Letters represent statistically similar groups based on statistical models assessing each behaviour as a function of either genus or species and corresponding post hoc analysis (*p* < 0.05). Here, the first row of coloured letters depicts statistically similar groups amongst the three genera *Aedes* (red), *Anopheles* (blue) and *Culex* (green), whereas the second row comprising black letters depicts statistically similar groups amongst all 13 tested species/strains. (a) The horizontal, dashed line at the 20‐min‐mark visualises the end of the 20‐min‐long trial period, with data points above this line depicting individuals which did not move throughout the entire trial. (c) The horizontal, dashed line at the 20‐min‐mark visualises the maximum time a pupa could have remained stationary within the 20‐min‐long trial period. (a–h) Sample size and sex ratio of each species as presented in Figure 1.

We established significant differences in the pupal swimming activity and performance across all tested mosquito species. Throughout the 20‐min‐long observation period, activity over time varied strongly between species (GLM: *χ* ^2^_12_ = 2510.40, *p* < 0.001), with *Aedes* pupae displaying continuous swimming behaviour across the entire arena, whereas pupae of the genera *Anopheles* and *Culex* showed comparably lower activity levels along with prolonged phases of inactivity (Figure 1). On genus level, *Aedes* pupae initiated their swimming activity earlier (Figure 2a), showed higher initial activity (Figure 2b), spent less time stationary (Figure 2c), displayed a higher number of movement bouts (Figure 2d), covered larger swimming distances (Figure 2e) and explored larger areas of the arena (Figure 2f) than both *Anopheles* and *Culex* pupae (all pairwise comparisons: *p* < 0.001, Tables A1 and A2). These behaviours did not differ between *Anopheles* and *Culex* (all pairwise comparisons: *p* > 0.050, Table A2), with the exception of initial activity, which was higher in *Anopheles* than in *Culex* (*t*_345_ = 1.99, *p* = 0.048). In contrast to these activity measurements, performance‐related traits showed an opposite pattern. Here, *Culex* pupae achieved higher swimming speeds and greater displacements with each movement bout than *Anopheles* pupae, whereas *Aedes* pupae exhibited the slowest swimming speeds and shortest displacements amongst the three genera (Figure 2g,h; Table A2).

**FIGURE 3:**
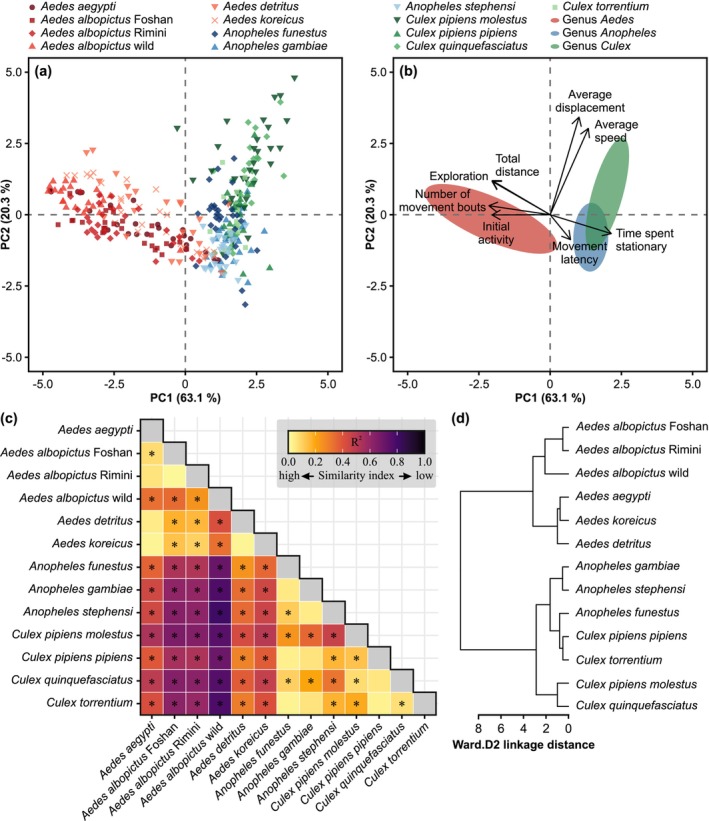
Similarities and differences in pupal swimming behaviour between species and genera. (a, b) Principal component analysis (PCA) plots of the eight analysed behavioural variables describing pupal swimming, based on obtained Euclidean distance matrix. (a) depicts individual datapoints in space, while (b) displays vector arrows indicating the direction and contribution of the eight behavioural variables, along with ellipses representing 68% confidence regions (corresponding approximately to one standard deviation) for each genus (red = *Aedes*, blue = *Anopheles*, green = *Culex*). (c) Heatmap illustrating behavioural similarities amongst the tested species. Lower *R* ^*2*^ values indicate more similar species, whereas higher *R* ^2^ values indicate greater dissimilarity, with asterisks denoting statistically dissimilar species (*p* < 0.050). (d) Hierarchical clustering dendrogram based on Euclidean distance matrix, displaying the grouping of species according to similarity in swimming behaviour. Branch lengths represent dissimilarity between clusters, with shorter branch lengths indicating more similar behavioural profiles. (a–d) Sample size and sex ratio of each species as presented in Figure 1.

To further assess the degree to which the swimming behaviour of mosquito pupae is genus‐specific, we constructed a Euclidean distance matrix based on the eight analysed behaviours, showing significant differences at both species‐ (perMANOVA; *F* = 38.98, *p* < 0.001) and genus‐level (perMANOVA; *F* = 165.05, *p* < 0.001). A principal component analysis (PCA) of the behavioural variables revealed an evident clustering of individuals according to genus (Figure 3a,b). In particular, variation along PC1 primarily reflected overall swimming activity, with individuals showing high initial activity, frequent movement bouts, long swimming distances and strong explorative behaviour clustering at negative PC1 values, whereas individuals characterised by high movement latency and prolonged stationary phases clustered at positive PC1 values. In addition, PC2 captured variation in swimming performance, separating individuals exhibiting higher swimming speeds and greater displacements per movement bout (positive PC2 values) from slower moving individuals. Consistent with our initial observations, PC1 (LM: *F*_2_ = 415.96, *p* < 0.001), PC2 (LM: *F*_2_ = 49.61, *p* < 0.001) and the combined PC1‐PC2 space (MANOVA: *F*_2_ = 198.55, Wilks' λ = 0.21, *p* < 0.001) differed significantly amongst genera, confirming that *Aedes* pupae generally displayed higher activity levels than *Anopheles* and *Culex*, with the latter two genera excelling at swimming speeds and displacement (Table A3). Using the Euclidean distance matrix, we further created a behaviour similarity heatmap (Figure 3c), highlighting how similar or dissimilar the tested species were in their overall swimming activity and performance. This approach revealed low similarity levels in the swimming behaviour between all *Aedes* species and species of the genera *Anopheles* and *Culex*, which are further confirmed by a hierarchical clustering dendrogram (Figure 3d) showing a strong separation of the *Aedes* pupae from the *Anopheles* and *Culex* pupae. Taken together, these results demonstrate clear genus‐specific differences in pupal swimming behaviours, with *Aedes* generally characterised by high activity but slower swimming speeds and low displacements per movement bout, whereas *Anopheles* and in particular *Culex* exhibit lower activity but higher swimming performances.

In addition to these evident behavioural differences between the three mosquito genera, we also established behavioural variation between species within the same genus. For species of the genus *Aedes*, hierarchical clustering based on Euclidean distances revealed a distinct separation of the three *Ae. albopictus* strains from the other three tested species *Ae. aegypti*, *Ae. detritus* and *Ae. koreicus* (Figure 3d). Indeed, all three *Ae. albopictus* lines are characterised by comparatively higher activity levels, in particular in terms of their initial activity (Figure 2b) and total number of movement bouts (Figure 2d). However, we found that the wild‐collected pupae of this species spent less time stationary (Wild vs. Foshan: *z* = −4.62, *p* < 0.001; Wild vs. Rimini: *z* = −4.20, *p* < 0.001), exhibited a larger number of movement bouts (Wild vs. Foshan: *t*_355_ = 2.33, *p* = 0.029; Wild vs. Rimini: *t*_355_ = 2.36, *p* = 0.028), covered larger swimming distances (Wild vs. Foshan: *t*_335_ = 4.99, *p* < 0.001; Wild vs. Rimini: *t*_304_ = 4.33, *p* < 0.001) and explored a larger area of the arena (Wild vs. Foshan: *z* = 4.04, *p* = 0.001; Wild vs. Rimini: *z* = 3.20, *p* = 0.002) than both established laboratory strains (Foshan and Rimini), whereas the movement latency, initial activity, average speed and average displacement did not differ between either of those three *Ae. albopictus* strains (Table A2). While the overall swimming behaviour amongst the remaining three *Aedes* species *Ae. aegypti*, *Ae. detritus* and *Ae. koreicus* is generally similar (Figure 3c), *Ae. detritus* can be considered the *Aedes* species with the lowest swimming activity, highlighted by significantly longer stationary periods (Figure 2c), lower number of movement bouts (Figure 2d) and less arena exploration (Figure 2f) amongst all tested Aedes species (Table A2).

Focusing on the tested species of the genus *Anopheles*, *An. funestus*, *An. gambiae* and *An. stephensi* showed comparably little variation in their swimming activity (Figure 2a–f) yet differed strongly in their swimming performance (Table A2). In particular, *An. funestus* swam significantly faster (*An. funestus* vs. *An. gambiae*: *t*_320_ = 3.54, *p* < 0.001; *An. funestus* vs. *An. stephensi*: *t*_320_ = 6.37, *p* < 0.001) and covered significantly larger distances per movement bout (*An. funestus* vs. *An. gambiae*: *t*_320_ = 3.52, *p* < 0.001; *An. funestus* vs. *An. stephensi*: *t*_320_ = 6.19, *p* < 0.001) than the other two species, whereas *An. stephensi* displayed the slowest swimming speeds (*An. gambiae* vs. *An. stephensi*: *t*_320_ = 3.06, *p* = 0.004) and shortest average displacements (*An. gambiae* vs. *An. stephensi*: *t*_320_ = 2.88, *p* = 0.006) of these three *Anopheles* species (Figure 2g,h). Of the four tested *Culex* species, we found that *Cx. p. molestus*, *Cx. p. pipiens*, *Cx. quinquefasciatus* and *Cx. torrentium* did not differ in their swimming activity (Figure 2a–e, Table A2), with the exception of their exploratory behaviour where *Cx. p. molestus* explored a larger area than *Cx. quinquefasciatus* (*z* = 2.17, *p* = 0.044; Figure 2f). However, the four species differed in their swimming performance, with *Cx. p. molestus* and *Cx. quinquefasciatus* swimming on average faster than *Cx. p. pipiens* and *Cx. torrentium* (Figure 2g), and *Cx. p. molestus* also displaying further displacements with each movement bout than the other three species (Figure 2h).

### 3.2 Effect of Sex, Body Length and Water Temperature

We found little evidence that the sex of mosquito pupae contributes to differences in the swimming activity and performance. Across all tested species, we established no significant differences between male and female pupae for any of the eight analysed behaviours (Table A4), with the exception of the wild‐collected *Ae. albopictus* strain where females showed higher swimming speeds (LM: *F*_1_ = 54.03, *p* < 0.001) and larger displacement (GLM: *χ* ^2^_1_ = 50.61, *p* < 0.001) than males.

In terms of body length, none of the swimming activity parameters (movement latency, initial activity, time spent stationary, number of movement bouts, swimming distance and arena exploration) correlated with pupae body length for any of the tested species (Table A4). However, for six of the 13 tested mosquito strains (*Ae. aegypti*, *Ae. albopictus* Foshan, *Ae. albopictus* wild, *Ae. detritus*, *An. stephensi* and *Cx. p. molestus*) we found that with increasing body length pupae decreased their average swimming speed (Table A4). For three of these species (*Ae. aegypti*, *Ae. albopictus* wild, *Ae. detritus*), an additional negative correlation between body length and average displacement was established, demonstrating that with increasing body length pupae covered on average shorter distances per movement bout (Table A4).

**FIGURE A2:**
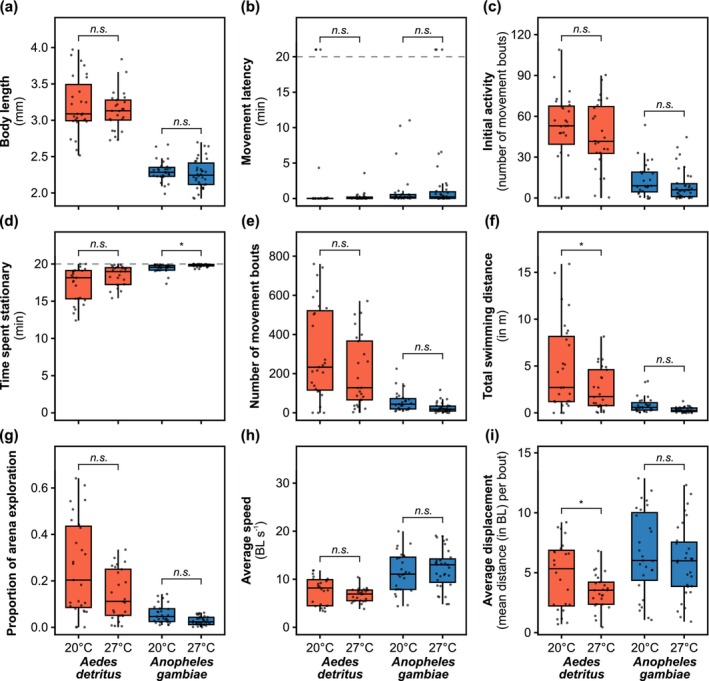
The effect of water temperature on pupal swimming behaviour. Our experimental design included both temperate and tropical mosquito species reared at different water temperatures. To determine whether differences in water temperature might explain some of the behavioural differences observed across all tested mosquito species, we recorded pupae of both the temperate species *Aedes detritus* (reared at 20°C) as well as the tropical species *Anopheles gambiae* (reared at 27°C) both 20°C and 27°C. (a) Body length of all *Ae. detritus* and *An. gambiae* pupae used for this analysis. (b–i) Species‐specific variation for the eight analysed behaviours at both 20°C and 27°C: (b) Movement latency (in min). The horizontal, dashed line at the 20‐min‐mark visualises the end of the 20‐min‐long trial period, with data points above this line depicting individuals which did not move throughout the entire trial. (c) Initial activity, defined as the number of movement bouts within the first 10% (2 min) of the trial. (d) Time spent stationary (in min). The horizontal, dashed line at the 20‐min‐mark visualises the maximum time a pupae could have remained stationary within the 20‐min‐long trial period. (e) Total number of observed movement bouts. (f) Total swimming distance (in m). (g) Proportion of arena exploration. (h) Average movement speed [in body length (BL) s^−1^]. (i) Average displacement, measured as the mean distance [in body length (BL)] covered per movement bout. (a–i) Each boxplot visualises the distribution for each species at a given temperature, whereas jittered grey points represent raw data. All boxplots represent the median (horizontal line) and quartiles (upper and lower boundaries) of a species' distribution, with the whiskers extending to the most extreme measurement within 1.5 times the interquartile range. Symbols above both temperature conditions of a species represent pairwise comparison based on post hoc analysis, indicated as *p < 0.05 or n.s., non‐significant.

We found little evidence that pupal swimming activity and performance were affected by water temperature and that pupae alter their swimming behaviour when exposed to temperatures other than their rearing conditions. While *Ae. detritus* covered less distance (*t*_109_ = 2.45, *p* = 0.016) and decreased their average bout length (*t*_103_ = 2.32, *p* = 0.022) at higher temperatures, and *An. gambiae* spent less time stationary at lower temperatures (*z* = −2.18, *p* = 0.029), we established no further temperature‐induced behavioural differences for *Ae. detritus* and *An. gambiae* (Figure A2; Table A5). As only one temperate and one tropical species were examined, we cannot fully exclude species‐specific responses to temperature. However, the limited effects observed suggest that the interspecific behavioural differences reported in this study are unlikely to be explained primarily by the different rearing temperatures used for temperate and tropical species.

## 4 Discussion

The *Aedes*, *Anopheles* and *Culex* pupae examined in this study displayed distinct differences in their swimming behaviour, with *Aedes* pupae swimming along the surface in a slow yet constant, exploratory fashion, whereas *Anopheles* and *Culex* pupae display a rather infrequent, burst‐like swimming style. Considering that juvenile mosquitoes occupy different, species‐specific habitat types (Becker et al. 2020), our established differences in pupal swimming behaviour may reflect behavioural adaptations associated with the habitat types they occupy. Indeed, many *Aedes* species, including *Ae. aegypti* (Getachew et al. 2015; Vijayakumar et al. 2014), *Ae. albopictus* (Vijayakumar et al. 2014) and *Ae. koreicus* (Montarsi et al. 2013), are considered container‐breeders, with adults frequently depositing their eggs in small, often man‐made, container‐like habitats such as buckets or tyres (Vezzani 2007). Compared with natural habitats, artificial containers are often characterised by fewer physical features to hide from predators and localised food resources (Vezzani 2007; Washburn 1995), resulting in larval aggregations which may provide antipredator benefits through dilution and encounter effects (Krause and Ruxton 2002). Although mosquito pupae do not feed and therefore do not need to seek food hotspots, we frequently observed *Aedes* pupae joining larval aggregations in our rearing trays. If *Aedes* pupae indeed seek shelter in larval aggregations to decrease their predation risk, the continuous swimming activity and vast exploratory behaviour of our solitarily tested *Aedes* pupae, particularly those belonging to the container‐breeding species, could potentially indicate a form of searching behaviour to find conspecifics.

In contrast to container‐breeding *Aedes*, most of our tested *Anopheles* and *Culex* species develop in natural, larger aquatic habitats such as ponds, streams, or temporary pools (Tandina et al. 2018; Gillies and De Meillon 1968; Liu et al. 2019), although *An. stephensi* is also known to exploit artificial containers (Taylor et al. 2024). Compared with these environments, artificial container habitats often support less diverse and less abundant predator communities, largely consisting of predaceous insects or their juvenile stages, which may contribute to the widespread use by container‐breeding mosquitoes (Vezzani 2007; Kitching 2000). In contrast, mosquito pupae developing in natural habitats are commonly exposed to a broader range of natural predators (Medlock and Snow 2008; Juliano 2009), including predaceous aquatic insects as well as various fish species which use visual or mechanical cues to locate prey. The higher predation pressure in these habitats may have favoured behavioural strategies in our tested *Anopheles* and *Culex* species that reduce the likelihood of predator detection, such as prolonged stationary phases and rapid movement patterns. In addition, natural habitats also frequently provide shelter opportunities through vegetation or other physical features which can reduce the predation risk of mosquito juvenile stages (Saha et al. 2012). In our experiments, many *Anopheles* and *Culex* pupae often decreased their swimming activity once they reached the arena wall and remained there stationary for long periods. It is thus possible that pupae of these two genera only show increased swimming activity when exposed to open water but reduce their activity once they reached shelter (Shuey et al. 1987), potentially indicating that their surface swimming behaviour acts as a mean of evasion rather than exploration. Interestingly, pupae of *Ae. detritus*, which in contrast to the other tested *Aedes* species are not container breeders but rather occupy large, natural salt marshes (Service 1968), showed the lowest activity levels but comparatively highest swimming speeds amongst all *Aedes* species. This might indicate that pupae of this species have somewhat adopted the potentially evasive swimming behaviour otherwise observed in natural habitat occupying *Anopheles* and *Culex* species.

Another striking difference between the tested species can be found in their body lengths, with the tested *Anopheles* species being significantly smaller than all *Aedes* and *Culex* species in this study. Across various animal taxa it has been shown that smaller individuals need to perform a larger number of movement cycles (e.g., steps) to cover the same absolute distance as larger individuals (Heglund et al. 1974). Therefore, we would expect *Anopheles* species to compensate for this size difference by performing higher number of movement bouts, or alternatively more energetic movement bouts covering larger distances with each bout, to cover the same swimming distance as *Aedes* or *Culex* pupae. However, the *Anopheles* species in our study generally displayed some of the lowest numbers of movement bouts amongst all species, and whilst performing on average further displacements than *Aedes* species, did not surpass the larger‐sized *Culex* species in their displacement rates. One explanation for this lack of compensation could be due to the potential evasive swimming behaviour outlined above, where *Anopheles* pupae only initiate swimming when disturbed or to reach sheltering opportunities. Alternatively, *Anopheles* might display less active swimming owing to potentially smaller energy reserves. As food uptake ceases with the end of the larval stage, pupal surface swimming relies entirely on available energy reserves (Ratnayake et al. 2023). Although studies investigating energy budgets of mosquito pupae are rare (Downer et al. 1976), especially on a comparative level between species, it has been shown for *Aedes*, *Anopheles* and *Culex* larvae that protein, lipid and carbohydrate reserves are positively correlated with body size (Timmermann and Briegel 1999). Assuming that this correlation also translates to pupal size, *Anopheles* pupae might display lower activity levels than *Aedes* and *Culex* due to limited energy reserves that restrain them from a more active or exploratory swimming style.

Despite our effort to quantify swimming behaviour under controlled laboratory conditions, it is important to acknowledge that our study may not fully establish all variation in pupal swimming behaviour potentially found in wild specimens. The shallow water depth (~6 mm) in our experimental setup constrained vertical movements, thereby preventing natural diving behaviour (Rodríguez‐Prieto et al. 2006; Awasthi et al. 2012; Futami et al. 2008; Chandrasegaran, Sriramamurthy, et al. 2020; Cordeschi et al. 2025). This was an intentionally chosen experimental design, as our objective was to quantify and compare surface swimming behaviour under standardised conditions across species. Consequently, our findings should be interpreted as reflecting interspecific differences in surface swimming rather than the full repertoire of aquatic locomotion. Moreover, many animal species display weakened or changed behaviours when kept for prolonged time in captivity (Elsbeth McPhee 2004). Indeed, we observed for *Ae. albopictus* that pupae from both laboratory strains (Foshan, maintained in captivity for 44 years (Chen et al. 2015); and Rimini, maintained in captivity for 21 years (Gomulski et al. 2018)) displayed reduced activity levels compared to wild‐collected pupae of this species, indicating that pupal swimming activity in captive mosquito populations might attenuate over time. In addition, our experimental setup lacked naturally occurring features, such as conspecifics, predators, complex physical structures, or sensory cues. Indeed, little is known about how natural features such as predator abundance, different types of vegetation and other environmental cues affect behavioural strategies of mosquito pupae. A promising avenue for future research, therefore, is to assess pupal swimming behaviour in natural settings to understand how natural pressures and available sensory information shape pupal behavioural strategies.

Another limitation of this study is the taxonomic breadth of the sampled species. Although we examined 11 mosquito species spanning the genera *Aedes*, *Anopheles* and *Culex*, several species within these genera belong to closely related groups, and overall, they represent only a small fraction of the approximately 3700 described mosquito species worldwide (Wilkerson et al. 2021). Therefore, the behavioural patterns reported here should be interpreted as evidence for genus‐specific differences amongst the sampled species rather than as universal characteristics of entire genera.

A more in‐depth investigation of pupal swimming behaviour in natural environments may further allow us to improve the development of mosquito control strategies. As mosquitoes are vectors of many life‐threatening diseases (Weaver et al. 2018), a large body of research is attributed to the development and refinement of approaches to limit mosquito populations and minimise or eliminate vector‐induced diseases (Wilson et al. 2020; Godfray 2013). In addition to control strategies for adults, multiple measures targeted to mosquito juvenile stages have also been developed (Lawler 2017). While some of these measures rely on ingestion, thereby becoming ineffective once the pupal life stage is reached, other strategies remain effective at both larval and pupal stages as they rely on different forms of habitat modifications (Lawler 2017). For example, the application of surface oils or monomolecular films is used to suffocate both larvae and pupae by preventing them from breathing (Nayar and Ali 2003). Alternatively, biological predators of mosquito juvenile stages can be introduced into aquatic habitats to reduce population sizes (Shaalan and Canyon 2009; Bhattacharjee et al. 2008; Griffin and Knight 2012). Our study showed that mosquito pupae can swim large distances, with some individuals covering approximately 15 m in less than 20 min, which suggests that pupae might be able to outswim locally applied control measures, thereby reducing their effectiveness. A better knowledge of pupal behavioural strategies in their natural environments, in particular how they use their distinctive ability to actively move through their aquatic habitats, would therefore be beneficial for improving pupae‐targeted control strategies and understanding how and where best to apply them.

In conclusion, our study revealed distinct, genus‐specific differences in the surface swimming activity and performance of mosquito pupae, an often overlooked but important life stage of the most devastating disease vector worldwide. In particular, pupae of the genus *Aedes* are characterised by slow but frequent movement bouts and high explorative behaviour, whereas *Anopheles* and *Culex* display fewer yet faster and longer movement bouts, suggesting that their swimming behaviour could be evasive rather than exploratory. Understanding how these differences are tailored by environmental pressures and habitat types will not only allow us to better understand the ecology of this heavily studied group of insects but might also be used to develop and improve mosquito control strategies to prevent disease transmission.

## Author Contributions

**Christian Drerup:** conceptualization (equal), data curation (lead), formal analysis (lead), investigation (lead), methodology (lead), software (lead), validation (lead), visualization (lead), writing – original draft (lead), writing – review and editing (equal). **Mingyue Feng:** investigation (supporting), validation (supporting), writing – review and editing (equal). **Jet S. Griep:** resources (supporting), writing – review and editing (equal). **Kyle J. Walker:** resources (supporting), writing – review and editing (equal). **Marta Villa:** resources (supporting), writing – review and editing (equal). **Amir Atapour‐Abarghouei:** resources (supporting), writing – review and editing (equal). **Sara Epis:** resources (supporting), writing – review and editing (equal). **Marcus S. C. Blagrove:** conceptualization (equal), funding acquisition (supporting), methodology (supporting), resources (supporting), writing – review and editing (equal). **Olena Riabinina:** conceptualization (equal), data curation (supporting), funding acquisition (lead), methodology (equal), project administration (lead), resources (lead), supervision (lead), validation (supporting), writing – review and editing (equal).

## Funding

This work was supported by a research grant to O.R. from the Leverhulme Trust (RPG‐2024‐151). M.F. was supported by a China Scholarship Council (Grant No. 202406350082). J.S.G. and K.J.W. were supported by the Biotechnology and Biological Sciences Research Council (BBSRC; BB/T008695/1) through the Newcastle, Liverpool, Durham Doctoral Training Program (NLD DTP; project references: J.S.G.—2749566; K.J.W.—2928822).

## Conflicts of Interest

The authors declare no conflicts of interest.

## Data Availability

Data and code are available from the Zenodo repository: https://doi.org/10.5281/zenodo.21130701 (Drerup 2026).

## Appendix A

**TABLE A1:** Output of the statistical analysis investigating variation in the eight behavioural variables on genus‐ and species‐level.

Behaviour	Model type	Response variable	*F*/*χ* ^2^	df	*p*
Movement latency	LM (log‐transformed)	Genus	*F* = 56.76	2	< 0.001
		Species	*F* = 10.49	12	< 0.001
Initial activity	LM (sqrt‐transformed)	Genus	*F* = 271.08	2	< 0.001
		Species	*F* = 59.52	12	< 0.001
Time spent stationary	GLM (family = beta)	Genus	*χ* ^2^ = 230.54	2	< 0.001
		Species	*χ* ^2^ = 362.56	12	< 0.001
Number of movement bouts	LM (sqrt‐transformed)	Genus	*F* = 382.03	2	< 0.001
		Species	*F* = 93.71	12	< 0.001
Total swimming distance	LM (sqrt‐transformed)	Genus	*F* = 221.22	2	< 0.001
		Species	*F* = 49.38	12	< 0.001
Arena exploration	GLM (family = beta‐binomial)	Genus	*χ* ^2^ = 211.20	2	< 0.001
		Species	*χ* ^2^ = 266.81	12	< 0.001
Average speed	LM (log‐transformed)	Genus	*F* = 198.50	2	< 0.001
		Species	*F* = 46.26	12	< 0.001
Average displacement	GLM (family = gamma)	Genus	*χ* ^2^ = 237.13	2	< 0.001
		Species	*χ* ^2^ = 475.78	12	< 0.001

**TABLE A2:** Post hoc analysis of the pupal swimming behaviour analysis. Estimated marginal means and statistical subsets for each behaviour on both species‐ and genus‐level.

Behaviour	Category	Species/genus	Emmean	SE	df	Lower CI	Upper CI	Subset
Movement latency	Species	*Aedes aegypti*	−5.03	0.42	320	−6.25	−3.18	a
		*Aedes albopictus Foshan*	−4.98	0.42	320	−6.19	−3.76	a
		*Aedes albopictus Rimini*	−4.59	0.42	320	−5.80	−3.37	ab
		*Aedes albopictus wild*	−5.45	0.42	320	−6.67	−4.24	a
		*Aedes detritus*	−4.51	0.44	320	−5.77	−3.24	ab
		*Aedes koreicus*	−4.54	0.43	320	−5.78	−3.30	ab
		*Anopheles funestus*	−3.39	0.40	320	−4.56	−2.21	bc
		*Anopheles gambiae*	−1.97	0.38	320	−3.09	−0.86	d
		*Anopheles stephensi*	−2.53	0.42	320	−3.74	−1.31	cd
		*Culex pipiens molestus*	−1.99	0.37	320	−3.07	−0.91	d
		*Culex pipiens pipiens*	−2.06	0.55	320	−3.66	−0.46	cd
		*Culex quinquefasciatus*	−2.14	0.38	320	−3.24	−1.05	d
		*Culex torrentium*	−2.72	0.55	320	−4.32	−1.12	cd
	Genus	*Aedes*	−4.86	0.17	330	−5.20	−4.52	a
		*Anopheles*	−2.61	0.23	330	−3.06	−2.15	b
		*Culex*	−2.17	0.22	330	−2.60	−1.74	b
Initial activity	Species	*Aedes aegypti*	7.58	0.39	335	6.45	8.71	c
		*Aedes albopictus Foshan*	9.69	0.39	335	8.56	10.82	d
		*Aedes albopictus Rimini*	9.24	0.39	335	8.11	10.37	d
		*Aedes albopictus wild*	9.60	0.39	335	8.47	10.73	d
		*Aedes detritus*	6.50	0.38	335	5.39	7.61	c
		*Aedes koreicus*	7.33	0.39	335	6.19	8.46	c
		*Anopheles funestus*	4.09	0.36	335	3.04	5.14	b
		*Anopheles gambiae*	2.46	0.34	335	1.47	3.45	a
		*Anopheles stephensi*	3.31	0.39	335	2.18	4.44	ab
		*Culex pipiens molestus*	2.67	0.35	335	1.67	3.68	a
		*Culex pipiens pipiens*	2.38	0.48	335	0.98	3.78	a
		*Culex quinquefasciatus*	2.59	0.34	335	1.60	3.58	a
		*Culex torrentium*	2.87	0.48	335	1.47	4.27	ab
	Genus	*Aedes*	8.31	0.17	345	7.90	8.72	c
		*Anopheles*	3.25	0.23	345	2.70	3.80	b
		*Culex*	2.63	0.22	345	2.11	3.15	a
Time spent stationary	Species	*Aedes aegypti*	1.32	0.13	Inf	0.95	1.69	cd
		*Aedes albopictus Foshan*	0.99	0.12	Inf	0.64	1.34	bc
		*Aedes albopictus Rimini*	0.91	0.12	Inf	0.57	1.25	b
		*Aedes albopictus wild*	0.23	0.11	Inf	−0.09	0.55	a
		*Aedes detritus*	2.36	0.16	Inf	1.89	2.83	e
		*Aedes koreicus*	1.71	0.14	Inf	1.30	2.11	d
		*Anopheles funestus*	3.19	0.18	Inf	2.67	3.71	f
		*Anopheles gambiae*	3.49	0.18	Inf	2.98	4.00	f
		*Anopheles stephensi*	3.22	0.19	Inf	2.67	3.78	f
		*Culex pipiens molestus*	3.21	0.17	Inf	2.71	3.70	f
		*Culex pipiens pipiens*	3.46	0.24	Inf	2.77	4.16	f
		*Culex quinquefasciatus*	3.46	0.18	Inf	2.96	3.97	f
		*Culex torrentium*	3.47	0.24	Inf	2.77	4.16	f
	Genus	*Aedes*	1.21	0.06	Inf	1.05	1.36	a
		*Anopheles*	2.97	0.12	Inf	2.69	3.26	b
		*Culex*	3.03	0.12	Inf	2.75	3.30	b
Number of movement bouts	Species	*Aedes aegypti*	20.51	0.96	335	17.72	23.30	d
		*Aedes albopictus Foshan*	24.58	0.96	335	21.79	27.37	e
		*Aedes albopictus Rimini*	24.54	0.96	335	21.75	27.33	e
		*Aedes albopictus wild*	27.74	0.96	335	24.95	30.53	f
		*Aedes detritus*	15.59	0.94	335	12.86	18.33	c
		*Aedes koreicus*	18.39	0.96	335	15.60	21.18	cd
		*Anopheles funestus*	7.06	0.89	335	4.47	9.66	b
		*Anopheles gambiae*	4.38	0.84	335	1.94	6.82	a
		*Anopheles stephensi*	6.40	0.96	335	3.61	9.19	ab
		*Culex pipiens molestus*	5.19	0.85	335	2.72	7.67	ab
		*Culex pipiens pipiens*	4.13	1.19	335	0.68	7.58	ab
		*Culex quinquefasciatus*	4.29	0.84	335	1.85	6.73	a
		*Culex torrentium*	4.43	1.19	335	0.98	7.88	ab
	Genus	*Aedes*	21.85	0.45	345	20.78	22.93	b
		*Anopheles*	5.86	0.59	345	4.43	7.28	a
		*Culex*	4.58	0.56	345	3.24	5.93	a
Swimming distance	Species	*Aedes aegypti*	68.40	4.13	335	56.36	80.40	de
		*Aedes albopictus Foshan*	72.30	4.13	335	60.30	84.30	de
		*Aedes albopictus Rimini*	76.20	4.13	335	61.14	88.20	e
		*Aedes albopictus wild*	101.40	4.13	335	89.40	113.40	f
		*Aedes detritus*	60.00	4.05	335	48.25	71.80	d
		*Aedes koreicus*	71.10	4.13	335	59.05	83.10	de
		*Anopheles funestus*	30.00	3.84	335	18.77	41.10	bc
		*Anopheles gambiae*	15.70	3.61	335	5.16	26.20	a
		*Anopheles stephensi*	19.00	4.13	335	6.94	31.00	ab
		*Culex pipiens molestus*	31.20	3.66	335	20.57	41.90	c
		*Culex pipiens pipiens*	20.10	5.10	335	5.25	35.00	abc
		*Culex quinquefasciatus*	22.30	3.61	335	11.83	32.80	abc
		*Culex torrentium*	20.90	5.10	335	6.08	35.80	abc
	Genus	*Aedes*	74.80	1.82	345	70.40	79.20	b
		*Anopheles*	21.40	2.41	345	15.60	27.20	a
		*Culex*	24.60	2.27	345	19.20	30.10	a
Exploration	Species	*Aedes aegypti*	0.29	0.03	Inf	0.21	0.37	d
		*Aedes albopictus Foshan*	0.30	0.03	Inf	0.22	0.38	d
		*Aedes albopictus Rimini*	0.33	0.03	Inf	0.25	0.42	d
		*Aedes albopictus wild*	0.47	0.03	Inf	0.38	0.57	e
		*Aedes detritus*	0.18	0.02	Inf	0.13	0.26	c
		*Aedes koreicus*	0.30	0.03	Inf	0.22	0.39	d
		*Anopheles funestus*	0.09	0.01	Inf	0.06	0.14	ab
		*Anopheles gambiae*	0.06	0.01	Inf	0.04	0.10	a
		*Anopheles stephensi*	0.07	0.01	Inf	0.04	0.11	ab
		*Culex pipiens molestus*	0.10	0.01	Inf	0.07	0.15	b
		*Culex pipiens pipiens*	0.07	0.01	Inf	0.04	0.13	ab
		*Culex quinquefasciatus*	0.06	0.01	Inf	0.04	0.10	a
		*Culex torrentium*	0.07	0.01	Inf	0.03	0.12	ab
	Genus	*Aedes*	−0.81	0.06	Inf	−0.96	−0.66	b
		*Anopheles*	−2.47	0.11	Inf	−2.74	−2.21	a
		*Culex*	−2.41	0.10	Inf	−2.66	−2.16	a
Average speed	Species	*Aedes aegypti*	1.81	0.06	320	1.62	1.99	abc
		*Aedes albopictus Foshan*	1.78	0.06	320	1.60	1.96	abc
		*Aedes albopictus Rimini*	1.71	0.06	320	1.53	1.89	a
		*Aedes albopictus wild*	1.73	0.06	320	1.55	1.92	ab
		*Aedes detritus*	1.94	0.07	320	1.75	2.13	c
		*Aedes koreicus*	1.91	0.06	320	1.73	2.10	bc
		*Anopheles funestus*	2.73	0.06	320	2.56	2.91	f
		*Anopheles gambiae*	2.44	0.06	320	2.27	2.60	e
		*Anopheles stephensi*	2.18	0.06	320	1.99	2.36	d
		*Culex pipiens molestus*	2.81	0.06	320	2.65	2.97	f
		*Culex pipiens pipiens*	2.47	0.08	320	2.23	2.71	e
		*Culex quinquefasciatus*	2.73	0.06	320	2.56	2.89	f
		*Culex torrentium*	2.39	0.08	320	2.15	2.63	de
	Genus	*Aedes*	1.81	0.03	330	1.74	1.88	a
		*Anopheles*	2.46	0.04	330	2.36	2.55	b
		*Culex*	2.66	0.04	330	2.58	2.75	c
Average displacement	Species	*Aedes aegypti*	1.03	0.08	320	0.79	1.26	a
		*Aedes albopictus Foshan*	0.93	0.08	320	0.69	1.17	a
		*Aedes albopictus Rimini*	0.91	0.08	320	0.67	1.15	a
		*Aedes albopictus wild*	1.14	0.08	320	0.90	1.38	a
		*Aedes detritus*	1.57	0.08	320	1.32	1.82	bc
		*Aedes koreicus*	1.40	0.08	320	1.16	1.64	b
		*Anopheles funestus*	2.16	0.08	320	1.93	2.39	e
		*Anopheles gambiae*	1.78	0.08	320	1.56	2.00	cd
		*Anopheles stephensi*	1.45	0.08	320	1.21	1.69	b
		*Culex pipiens molestus*	2.44	0.07	320	2.23	2.66	f
		*Culex pipiens pipiens*	1.90	0.11	320	1.59	2.22	de
		*Culex quinquefasciatus*	2.08	0.07	320	1.86	2.29	e
		*Culex torrentium*	2.05	0.11	320	1.73	2.36	de
	Genus	*Aedes*	1.19	0.04	330	1.09	1.29	a
		*Anopheles*	1.84	0.06	330	1.71	1.98	b
		*Culex*	2.19	0.05	330	2.07	2.32	c

**TABLE A3:** Post hoc analysis of the genus‐specific contribution to PC1, PC2 and the PC1–PC2 space of our principal component analysis.

Category	Genus	emmean	SE	df	Lower CI	Upper CI	Subset
PC1	*Aedes*	−2.05	0.10	330	−2.28	−1.81	a
	*Anopheles*	1.44	0.13	330	1.12	1.75	b
	*Culex*	2.01	0.12	330	1.71	2.31	c
PC2	*Aedes*	−0.77	0.12	330	−1.06	−0.48	a
	*Anopheles*	−0.11	0.09	330	−0.33	0.10	b
	*Culex*	0.87	0.12	330	0.60	1.15	c
PC1‐PC2 space	*Aedes*	−1.08	0.06	330	−1.22	−0.94	a
	*Anopheles*	0.33	0.08	330	0.15	0.52	b
	*Culex*	1.44	0.07	330	1.27	1.62	c

**TABLE A4:** Effect of pupal sex and size on tested behaviours.

Behaviour	Species	Sex	Size
Movement latency	*Aedes aegypti*	LM: *F*_1_ = 0.46, *p* = 0.506	LM: *F*_1_ = 1.09, *p* = 0.306
	*Aedes albopictus Foshan*	LM: *F*_1_ = 0.50, *p* = 0.485	LM: *F*_1_ = 0.13, *p* = 0.722
	*Aedes albopictus Rimini*	LM: *F*_1_ = 2.20, *p* = 0.152	LM: *F*_1_ = 3.63, *p* = 0.069
	*Aedes albopictus wild*	LM: *F*_1_ = 1.94, *p* = 0.177	LM: *F*_1_ = 0.04, *p* = 0.839
	*Aedes detritus*	LM: *F*_1_ = 0.03, *p* = 0.870	LM: *F*_1_ < 0.01, *p* = 0.982
	*Aedes koreicus*	LM: *F*_1_ = 2.52, *p* = 0.127	LM: *F*_1_ = 0.37, *p* = 0.549
	*Anopheles funestus*	LM: *F*_1_ = 2.40, *p* = 0.134	LM: *F*_1_ = 0.07, *p* = 0.787
	*Anopheles gambiae*	LM: *F*_1_ = 1.11, *p* = 0.301	LM: *F*_1_ = 0.66, *p* = 0.424
	*Anopheles stephensi*	LM: *F*_1_ = 0.12, *p* = 0.730	LM: *F*_1_ = 0.75, *p* = 0.395
	*Culex pipiens molestus*	LM: *F*_1_ = 0.09, *p* = 0.771	LM: *F*_1_ = 0.63, *p* = 0.433
	*Culex pipiens pipiens*	LM: *F*_1_ = 0.80, *p* = 0.388	LM: *F*_1_ = 0.30, *p* = 0.593
	*Culex quinquefasciatus*	LM: *F*_1_ = 0.01, *p* = 0.905	LM: *F*_1_ = 1.39, *p* = 0.248
	*Culex torrentium*	LM: *F*_1_ = 0.01, *p* = 0.915	LM: *F*_1_ = 0.01, *p* = 0.924
Initial activity	*Aedes aegypti*	LM: *F*_1_ = 0.81, *p* = 0.378	LM: *F*_1_ = 1.27, *p* = 0.272
	*Aedes albopictus Foshan*	LM: *F*_1_ = 3.39, *p* = 0.079	LM: *F*_1_ = 2.54, *p* = 0.124
	*Aedes albopictus Rimini*	LM: *F*_1_ = 2.29, *p* = 0.144	LM: *F*_1_ = 2.27, *p* = 0.146
	*Aedes albopictus wild*	LM: *F*_1_ = 2.17, *p* = 0.154	LM: *F*_1_ = 0.03, *p* = 0.869
	*Aedes detritus*	LM: *F*_1_ = 0.30, *p* = 0.587	LM: *F*_1_ = 0.70, *p* = 0.411
	*Aedes koreicus*	LM: *F*_1_ = 0.29, *p* = 0.594	LM: *F*_1_ < 0.01, *p* = 0.947
	*Anopheles funestus*	LM: *F*_1_ = 0.87, *p* = 0.359	LM: *F*_1_ = 3.03, *p* = 0.092
	*Anopheles gambiae*	LM: *F*_1_ = 0.47, *p* = 0.496	LM: *F*_1_ = 0.04, *p* = 0.848
	*Anopheles stephensi*	LM: *F*_1_ = 1.57, *p* = 0.223	LM: *F*_1_ = 0.68, *p* = 0.417
	*Culex pipiens molestus*	LM: *F*_1_ = 1.60, *p* = 0.215	LM: *F*_1_ = 3.43, *p* = 0.074
	*Culex pipiens pipiens*	LM: *F*_1_ = 4.49, *p* = 0.052	LM: *F*_1_ = 0.01, *p* = 0.940
	*Culex quinquefasciatus*	LM: *F*_1_ = 0.06, *p* = 0.815	LM: *F*_1_ = 0.64, *p* = 0.430
	*Culex torrentium*	LM: *F*_1_ = 0.03, *p* = 0.877	LM: *F*_1_ = 0.23, *p* = 0.637
Time spent swimming	*Aedes aegypti*	GLM: *χ* ^2^_1_ = 1.44, *p* = 0.231	GLM: *χ* ^2^_1_ = 0.89, *p* = 0.344
	*Aedes albopictus Foshan*	GLM: *χ* ^2^_1_ = 1.26, *p* = 0.263	GLM: *χ* ^2^_1_ = 2.09, *p* = 0.149
	*Aedes albopictus Rimini*	GLM: *χ* ^2^_1_ = 1.15, *p* = 0.283	GLM: *χ* ^2^_1_ = 0.10, *p* = 0.754
	*Aedes albopictus wild*	GLM: *χ* ^2^_1_ = 0.17, *p* = 0.684	GLM: *χ* ^2^_1_ = 0.04, *p* = 0.832
	*Aedes detritus*	GLM: *χ* ^2^_1_ < 0.01, *p* = 0.991	GLM: *χ* ^2^_1_ = 2.21, *p* = 0.137
	*Aedes koreicus*	GLM: *χ* ^2^_1_ = 0.95, *p* = 0.330	GLM: *χ* ^2^_1_ = 0.23, *p* = 0.629
	*Anopheles funestus*	GLM: *χ* ^2^_1_ = 0.25, *p* = 0.615	GLM: *χ* ^2^_1_ = 1.66, *p* = 0.198
	*Anopheles gambiae*	GLM: *χ* ^2^_1_ = 0.01, *p* = 0.941	GLM: *χ* ^2^_1_ = 0.21, *p* = 0.651
	*Anopheles stephensi*	GLM: *χ* ^2^_1_ = 1.02, *p* = 0.312	GLM: *χ* ^2^_1_ = 0.28, *p* = 0.599
	*Culex pipiens molestus*	GLM: *χ* ^2^_1_ = 0.33, *p* = 0.567	GLM: *χ* ^2^_1_ = 0.88, *p* = 0.348
	*Culex pipiens pipiens*	GLM: *χ* ^2^_1_ = 2.78, *p* = 0.096	GLM: *χ* ^2^_1_ = 1.63, *p* = 0.202
	*Culex quinquefasciatus*	GLM: *χ* ^2^_1_ = 0.18, *p* = 0.669	GLM: *χ* ^2^_1_ = 1.29, *p* = 0.257
	*Culex torrentium*	GLM: *χ* ^2^_1_ = 0.71, *p* = 0.401	GLM: *χ* ^2^_1_ = 1.57, *p* = 0.211
Number of movement bouts	*Aedes aegypti*	LM: *F*_1_ = 1.82, *p* = 0.190	LM: *F*_1_ = 2.01, *p* = 0.170
	*Aedes albopictus Foshan*	LM: *F*_1_ = 1.26, *P* = 0.272	LM: *F*_1_ = 3.87, *p* = 0.061
	*Aedes albopictus Rimini*	LM: *F*_1_ = 1.83, *p* = 0.189	LM: *F*_1_ = 1.11, *p* = 0.302
	*Aedes albopictus wild*	LM: *F*_1_ = 4.18, *p* = 0.052	LM: *F*_1_ = 0.33, *p* = 0.572
	*Aedes detritus*	LM: *F*_1_ < 0.01, *p* = 0.924	LM: *F*_1_ = 0.82, *p* = 0.374
	*Aedes koreicus*	LM: *F*_1_ < 0.01, *p* = 0.981	LM: *F*_1_ = 0.03, *p* = 0.865
	*Anopheles funestus*	LM: *F*_1_ = 0.09, *p* = 0.763	LM: *F*_1_ = 0.53, *p* = 0.472
	*Anopheles gambiae*	LM: *F*_1_ = 0.24, *p* = 0.631	LM: *F*_1_ = 0.57, *p* = 0.458
	*Anopheles stephensi*	LM: *F*_1_ = 0.62, *p* = 0.441	LM: *F*_1_ = 0.99, *p* = 0.331
	*Culex pipiens molestus*	LM: *F*_1_ = 0.06, *p* = 0.803	LM: *F*_1_ = 2.26, *p* = 0.143
	*Culex pipiens pipiens*	LM: *F*_1_ = 1.80, *p* = 0.201	LM: *F*_1_ = 0.40, *p* = 0.536
	*Culex quinquefasciatus*	LM: *F*_1_ = 0.05, *p* = 0.826	LM: *F*_1_ = 0.32, *p* = 0.576
	*Culex torrentium*	LM: *F*_1_ = 0.23, *p* = 0.639	LM: *F*_1_ = 0.41, *p* = 0.532
Distance travelled	*Aedes aegypti*	LM: *F*_1_ = 2.54, *p* = 0.125	LM: *F*_1_ = 1.55, *p* = 0.226
	*Aedes albopictus Foshan*	LM: *F*_1_ = 1.24, *p* = 0.276	LM: *F*_1_ = 0.72, *p* = 0.407
	*Aedes albopictus Rimini*	LM: *F*_1_ = 0.71, *P* = 0.407	LM: *F*_1_ = 1.32, *p* = 0.261
	*Aedes albopictus wild*	LM: *F*_1_ = 0.75, *p* = 0.396	LM: *F*_1_ = 0.02, *p* = 0.896
	*Aedes detritus*	LM: *F*_1_ < 0.01, *p* = 0.973	LM: *F*_1_ = 3.70, *p* = 0.066
	*Aedes koreicus*	LM: *F*_1_ = 0.01, *p* = 0.942	LM: *F*_1_ = 0.23, *p* = 0.633
	*Anopheles funestus*	LM: *F*_1_ = 0.01, *p* = 0.928	LM: *F*_1_ = 2.07, *p* = 0.161
	*Anopheles gambiae*	LM: *F*_1_ < 0.01, *p* = 0.984	LM: *F*_1_ = 0.10, *p* = 0.749
	*Anopheles stephensi*	LM: *F*_1_ = 0.07, *p* = 0.787	LM: *F*_1_ = 1.91, *p* = 0.180
	*Culex pipiens molestus*	LM: *F*_1_ = 0.10, *p* = 0.758	LM: *F*_1_ = 1.88, *p* = 0.181
	*Culex pipiens pipiens*	LM: *F*_1_ = 1.78, *p* = 0.204	LM: *F*_1_ = 1.50, *p* = 0.241
	*Culex quinquefasciatus*	LM: *F*_1_ = 0.11, *p* = 0.746	LM: *F*_1_ = 0.79, *p* = 0.381
	*Culex torrentium*	LM: *F*_1_ = 0.29, *p* = 0.596	LM: *F*_1_ = 0.10, *p* = 0.755
Exploration	*Aedes aegypti*	GLM: *χ* ^2^_1_ = 1.74, *p* = 0.187	GLM: *χ* ^2^_1_ = 0.72, *p* = 0.397
	*Aedes albopictus Foshan*	GLM: *χ* ^2^_1_ = 0.30, *p* = 0.584	GLM: *χ* ^2^_1_ = 1.23, *p* = 0.267
	*Aedes albopictus Rimini*	GLM: *χ* ^2^_1_ = 0.95, *p* = 0.330	GLM: *χ* ^2^_1_ = 0.51, *p* = 0.475
	*Aedes albopictus wild*	GLM: *χ* ^2^_1_ = 0.02, *p* = 0.892	GLM: *χ* ^2^_1_ = 0.04, *p* = 0.836
	*Aedes detritus*	GLM: *χ* ^2^_1_ = 0.01, *p* = 0.920	GLM: *χ* ^2^_1_ = 3.28, *p* = 0.070
	*Aedes koreicus*	GLM: *χ* ^2^_1_ = 0.30, *p* = 0.581	GLM: *χ* ^2^_1_ = 0.03, *p* = 0.874
	*Anopheles funestus*	GLM: *χ* ^2^_1_ = 0.22, *p* = 0.642	GLM: *χ* ^2^_1_ = 0.70, *p* = 0.404
	*Anopheles gambiae*	GLM: *χ* ^2^_1_ = 0.07, *p* = 0.786	GLM: *χ* ^2^_1_ = 2.24, *p* = 0.135
	*Anopheles stephensi*	GLM: *χ* ^2^_1_ = 1.21, *p* = 0.271	GLM: *χ* ^2^_1_ = 0.15, *p* = 0.700
	*Culex pipiens molestus*	GLM: *χ* ^2^_1_ = 0.25, *p* = 0.618	GLM: *χ* ^2^_1_ = 0.61, *p* = 0.436
	*Culex pipiens pipiens*	GLM: *χ* ^2^_1_ = 1.97, *p* = 0.160	GLM: *χ* ^2^_1_ = 2.02, *p* = 0.155
	*Culex quinquefasciatus*	GLM: *χ* ^2^_1_ = 0.39, *p* = 0.530	GLM: *χ* ^2^_1_ = 1.87, *p* = 0.171
	*Culex torrentium*	GLM: *χ* ^2^_1_ = 0.60, *p* = 0.438	GLM: *χ* ^2^_1_ = 1.41, *p* = 0.236
Average speed	*Aedes aegypti*	LM: *F*_1_ = 0.03, *p* = 0.876	**LM: *F***_**1**_ **= 12.88, *p* = 0.002**
	*Aedes albopictus Foshan*	LM: *F*_1_ = 0.09, *p* = 0.772	**LM: *F***_**1**_ **= 8.70, *p* = 0.007**
	*Aedes albopictus Rimini*	LM: *F*_1_ = 0.29, *p* = 0.595	LM: *F*_1_ = 2.32, *p* = 0.141
	*Aedes albopictus wild*	**LM: *F***_**1**_ **= 54.03, *p* < 0.001**	**LM: *F***_**1**_ **= 101.36, *p* < 0.001**
	*Aedes detritus*	LM: *F*_1_ = 0.05, *p* = 0.818	**LM: *F***_**1**_ **= 20.86, *p* < 0.001**
	*Aedes koreicus*	LM: *F*_1_ = 0.65, *p* = 0.427	LM: *F*_1_ = 1.71, *p* = 0.205
	*Anopheles funestus*	LM: *F*_1_ = 0.94, *p* = 0.340	LM: *F*_1_ = 0.61, *p* = 0.441
	*Anopheles gambiae*	LM: *F*_1_ = 1.74, *p* = 0.197	LM: *F*_1_ = 1.01, *p* = 0.324
	*Anopheles stephensi*	LM: *F*_1_ = 2.74, *p* = 0.111	**LM: *F***_**1**_ **= 5.64, *p* = 0.026**
	*Culex pipiens molestus*	LM: *F*_1_ = 0.27, *p* = 0.610	**LM: *F***_**1**_ **= 4.17, *p* = 0.049**
	*Culex pipiens pipiens*	LM: *F*_1_ = 0.33, *p* = 0.571	LM: *F*_1_ = 0.15, *p* = 0.708
	*Culex quinquefasciatus*	LM: *F*_1_ = 3.46, *p* = 0.073	LM: *F*_1_ = 1.47, *p* = 0.234
	*Culex torrentium*	LM: *F*_1_ = 0.01, *p* = 0.933	LM: *F*_1_ = 2.08, *p* = 0.175
Average displacement	*Aedes aegypti*	GLM: *χ* ^2^_1_ = 1.43, *p* = 0.231	**GLM: *χ*** ^**2**^_**1**_ **= 3.89, *p* = 0.049**
	*Aedes albopictus Foshan*	GLM: *χ* ^2^_1_ = 0.21, *p* = 0.643	GLM: *χ* ^2^_1_ = 0.17, *p* = 0.679
	*Aedes albopictus Rimini*	GLM: *χ* ^2^_1_ = 0.19, *p* = 0.662	GLM: *χ* ^2^_1_ = 1.00, *p* = 0.317
	*Aedes albopictus wild*	**GLM: *χ*** ^**2**^_**1**_ **= 50.61, *p* < 0.001**	**GLM: *χ*** ^**2**^_**1**_ **= 36.46, *p* < 0.001**
	*Aedes detritus*	GLM: *χ* ^2^_1_ = 1.46, *p* = 0.227	**GLM: *χ*** ^**2**^_**1**_ **= 17.62, *p* < 0.001**
	*Aedes koreicus*	GLM: *χ* ^2^_1_ = 0.08, *p* = 0.770	GLM: *χ* ^2^_1_ < 0.01, *p* = 0.953
	*Anopheles funestus*	GLM: *χ* ^2^_1_ = 0.99, *p* = 0.319	GLM: *χ* ^2^_1_ = 0.98, *p* = 0.321
	*Anopheles gambiae*	GLM: *χ* ^2^_1_ = 0.19, *p* = 0.666	GLM: *χ* ^2^_1_ = 0.46, *p* = 0.499
	*Anopheles stephensi*	GLM: *χ* ^2^_1_ = 0.42, *p* = 0.515	GLM: *χ* ^2^_1_ = 2.24, *p* = 0.134
	*Culex pipiens molestus*	GLM: *χ* ^2^_1_ = 0.15, *p* = 0.698	GLM: *χ* ^2^_1_ = 3.35, *p* = 0.067
	*Culex pipiens pipiens*	GLM: *χ* ^2^_1_ = 0.04, *p* = 0.839	GLM: *χ* ^2^_1_ < 0.01, *p* = 0.972
	*Culex quinquefasciatus*	GLM: *χ* ^2^_1_ = 0.09, *p* = 0.762	GLM: *χ* ^2^_1_ = 0.11, *p* = 0.742
	*Culex torrentium*	GLM: *χ* ^2^_1_ = 0.12, *p* = 0.728	GLM: *χ* ^2^_1_ = 0.02, *p* = 0.890

*Note:* Bold entries denote significant outcomes.

**TABLE A5:** Pairwise comparison for the temperature conditions.

Variable	Comparison	Estimate	SE	df	Ratio	*p*
Body length	*Ae. detritus* 20°C vs. *Ae. detritus* 27°C	0.07	0.07	109	*t* = 1.02	0.311
	*An. gambiae* 20°C vs. *An. gambiae* 27°C	0.04	0.07	109	*t* = 0.52	0.607
Movement latency	*Ae. detritus* 20°C vs. *Ae. detritus* 27°C	−0.97	0.66	103	*t* = −1.48	0.142
	*An. gambiae* 20°C vs. *An. gambiae* 27°C	−0.34	0.61	103	*t* = 0.56	0.576
Initial activity	*Ae. detritus* 20°C vs. *Ae. detritus* 27°C	0.11	0.63	109	*t* = 0.17	0.868
	*An. gambiae* 20°C vs. *An. gambiae* 27°C	0.70	0.58	109	*t* = 1.20	0.235
Time spent swimming	*Ae. detritus* 20°C vs. *Ae. detritus* 27°C	−0.13	0.24	inf	*z* = −0.56	0.577
	***An. gambiae* 20°C vs. *An. gambiae* 27°C**	**−0.53**	**0.24**	**inf**	***z* = 2.18**	**0.029**
Movement bouts	*Ae. detritus* 20°C vs. *Ae. detritus* 27°C	2.88	1.52	109	*t* = 1.90	0.061
	*An. gambiae* 20°C vs. *An. gambiae* 27°C	2.55	1.41	109	*t* = 1.81	0.074
Swimming distance	***Ae. detritus* 20°C vs. *Ae. detritus* 27°C**	**15.5**	**6.31**	**109**	***t* = 2.45**	**0.016**
	*An. gambiae* 20°C vs. *An. gambiae* 27°C	10.7	5.86	109	*t* = 1.82	0.071
Arena exploration	*Ae. detritus* 20°C vs. *Ae. detritus* 27°C	1.47	0.35	inf	*z* = 1.62	0.106
	*An. gambiae* 20°C vs. *An. gambiae* 27°C	1.46	0.35	inf	*z* = 1.55	0.120
Average speed	*Ae. detritus* 20°C vs. *Ae. detritus* 27°C	0.07	0.11	103	*t* = 0.66	0.512
	*An. gambiae* 20°C vs. *An. gambiae* 27°C	−0.07	0.10	103	*t* = 0.73	0.467
Average displacement	***Ae. detritus* 20°C vs. *Ae. detritus* 27°C**	**0.35**	**0.15**	**103**	***t* = 2.32**	**0.022**
	*An. gambiae* 20°C vs. *An. gambiae* 27°C	0.12	0.14	103	*t* = 0.87	0.388

*Note:* Bold entries denote significant outcomes.

## References

[ece374061-bib-0001] Awasthi, A. K. , C.‐H. Wu , and J.‐S. Hwang . 2012. “Diving as an Anti‐Predator Behavior in Mosquito Pupae.” Zoological Studies 51: 1225–1234.

[ece374061-bib-0002] Bahnck, C. M. , and D. M. Fonseca . 2006. “Rapid Assay to Identify the Two Genetic Forms of *Culex* (*Culex*) *Pipiens* L. (Dipteria: Culicidae) and Hybrid Populations.” American Journal of Tropical Medicine and Hygiene 75: 251–255. 10.4269/ajtmh.2006.75.2.0750251. 16896127

[ece374061-bib-0003] Bansal, P. , and S. Q. Sen . 2025. “Sexual Dimorphism in the Behaviour and Sensory Systems of Mosquitoes.” Current Opinion in Neurobiology 93: 103070. 10.1016/j.conb.2025.103070. 40609166

[ece374061-bib-0004] Bates, D. , M. Mächler , B. Bolker , and S. Walker . 2015. “Fitting Linear Mixed‐Effects Models Using lme4.” Journal of Statistical Software 67: 1–48. 10.18637/jss.v067.i01.

[ece374061-bib-0005] Becker, N. , D. Petrić , M. Zgomba , et al. 2020. “Key to Mosquito Fourth‐Instar Larvae.” In Mosquitoes: Identification, Ecology and Control, 143–167. Springer International Publishing.

[ece374061-bib-0006] Benjamini, Y. , and Y. Hochberg . 1995. “Controlling the False Discovery Rate: A Practical and Powerful Approach to Multiple Testing.” Journal of the Royal Statistical Society. Series B, Statistical Methodology 57: 289–300. 10.1111/j.2517-6161.1995.tb02031.x.

[ece374061-bib-0007] Bhattacharjee, I. , A. Ghosh , G. Chandra , and S. N. Chatterjee . 2008. “Mosquito Control by Larvivorous Fish.” Indian Journal of Medical Research 127: 13–27. 18316849

[ece374061-bib-0008] Brackenbury, J. 1999. “Regulation of Swimming in the *Culex pipiens* (Diptera, Culicidae) pupa: Kinematics and Locomotory Trajectories.” Journal of Experimental Biology 202: 2521–2529. 10.1242/jeb.202.18.2521. 10460739

[ece374061-bib-0009] Brooks, M. , K. Kristensen , K. van Benthem , et al. 2017. “glmmTMB Balances Speed and Flexibility Among Packages for Zero‐Inflated Generalized Linear Mixed Modeling.” R Journal 9: 378–400.

[ece374061-bib-0010] Chandrasegaran, K. , C. Lahondère , L. E. Escobar , and C. Vinauger . 2020. “Linking Mosquito Ecology, Traits, Behavior, and Disease Transmission.” Trends in Parasitology 36: 393–403. 10.1016/j.pt.2020.02.001. 32191853

[ece374061-bib-0011] Chandrasegaran, K. , R. Sriramamurthy , A. Singh , P. Ravichandran , and S. Quader . 2020. “Antipredatory Responses of Mosquito Pupae to Non‐Lethal Predation Threat‐Behavioral Plasticity Across Life‐History Stages.” Environmental Entomology 49: 1032–1040. 10.1093/ee/nvaa101. 32885816

[ece374061-bib-0012] Chen, X.‐G. , X. Jiang , J. Gu , et al. 2015. “Genome Sequence of the Asian Tiger Mosquito, *Aedes albopictus* , Reveals Insights Into Its Biology, Genetics, and Evolution.” Proc. Natl. Acad. Sci. USA 112: E5907–E5915. 10.1073/pnas.1516410112. 26483478 PMC4640774

[ece374061-bib-0013] Cordeschi, G. , V. Mastrantonio , R. Bisconti , N. Giardiello , D. Canestrelli , and D. Porretta . 2025. “Should I Dive or Should I Float? Behavioural Plasticity of *Aedes mariae* Pupae Under Predation Threat.” Parasites & Vectors 18: 224. 10.1186/s13071-025-06875-z. 40528222 PMC12175317

[ece374061-bib-0014] Downer, R. G. H. , J. H. Spring , and S. M. Smith . 1976. “Effect of an Insect Growth Regulator on Lipid and Carbohydrate Reserves of Mosquito Pupae (Diptera: Culicidae).” Canadian Entomologist 108: 627–630. 10.4039/Ent108627-6.

[ece374061-bib-0015] Drerup, C. 2026. “Data for ‘Distinct Swimming Behaviours in Pupae of *Aedes*, *Anopheles*, and *Culex* Mosquitoes’.” Zenodo. 10.5281/zenodo.21130701. PMC13402102 42516219

[ece374061-bib-0016] Egid, B. R. , M. Coulibaly , S. K. Dadzie , et al. 2022. “Review of the Ecology and Behaviour of *Aedes aegypti* and *Aedes albopictus* in Western Africa and Implications for Vector Control.” Current Research in Parasitology & Vector‐Borne Diseases 2: 100074. 10.1016/j.crpvbd.2021.100074. 35726222 PMC7612875

[ece374061-bib-0017] Elsbeth McPhee, M. 2004. “Generations in Captivity Increases Behavioral Variance: Considerations for Captive Breeding and Reintroduction Programs.” Biological Conservation 115: 71–77. 10.1016/S0006-3207(03)00095-8.

[ece374061-bib-0018] Folmer, O. , M. Black , W. Hoeh , R. Lutz , and R. Vrijenhoek . 1994. “DNA Primers for Amplification of Mitochondrial Cytochrome c Oxidase Subunit I From Diverse Metazoan Invertebrates.” Molecular Marine Biology and Biotechnology 3: 294–299. 7881515

[ece374061-bib-0019] Futami, K. , G. Sonye , P. Akweywa , S. Kaneko , and N. Minakawa . 2008. “Diving Behavior in *Anopheles gambiae* (Diptera: Culicidae): Avoidance of a Predacious Wolf Spider (Araneae: Lycosidae) in Relation to Life Stage and Water Depth.” Journal of Medical Entomology 45: 1050–1056. 10.1093/jmedent/45.6.1050. 19058628

[ece374061-bib-0020] Getachew, D. , H. Tekie , T. Gebre‐Michael , M. Balkew , and A. Mesfin . 2015. “Breeding Sites of *Aedes aegypti* : Potential Dengue Vectors in Dire Dawa, East Ethiopia.” Interdisciplinary Perspectives on Infectious Diseases 2015: 706276. 10.1155/2015/706276. 26435712 PMC4576013

[ece374061-bib-0021] Gillies, M. , and B. De Meillon . 1968. The Anophelinae of Africa South of the Sahara (Ethiopian Zoogeographical Region). South African Institute for Medical Research.

[ece374061-bib-0022] Godfray, H. C. J. 2013. “Mosquito Ecology and Control of Malaria.” Journal of Animal Ecology 82: 15–25. 10.1111/1365-2656.12003. 23148823

[ece374061-bib-0023] Gomulski, L. M. , M. Mariconti , A. Di Cosimo , et al. 2018. “The Nix Locus on the Male‐Specific Homologue of Chromosome 1 in *Aedes albopictus* Is a Strong Candidate for a Male‐Determining Factor.” Parasites & Vectors 11: 647. 10.1186/s13071-018-3215-8. 30583734 PMC6304787

[ece374061-bib-0024] Griffin, L. F. , and J. M. Knight . 2012. “A Review of the Role of Fish as Biological Control Agents of Disease Vector Mosquitoes in Mangrove Forests: Reducing Human Health Risks While Reducing Environmental Risk.” Wetlands Ecology and Management 20: 243–252. 10.1007/s11273-012-9248-4.

[ece374061-bib-0025] Hall, M. J. R. , and D. Martín‐Vega . 2019. “Visualization of Insect Metamorphosis.” Philosophical Transactions of the Royal Society, B: Biological Sciences 374: 20190071. 10.1098/rstb.2019.0071. PMC6711283 31438819

[ece374061-bib-0026] Hartig, F. 2022. “DHARMa: Residual Diagnostics for Hierarchical (Multi‐Level/Mixed) Regression Models. R Package Version 0.4.5.” https://cran.r‐project.org/package=DHARMa.

[ece374061-bib-0027] Hawkes, F. M. , and R. J. Hopkins . 2021. “The Mosquito: An Introduction.” In Mosquitopia, edited by M. Hall and D. Tamir , 16–31. Routledge. 36260714

[ece374061-bib-0028] Heglund, N. C. , C. R. Taylor , and T. A. McMahon . 1974. “Scaling Stride Frequency and Gait to Animal Size: Mice to Horses.” Science 186: 1112–1113. 10.1126/science.186.4169.1112. 4469699

[ece374061-bib-0029] Hesson, J. C. , J. O. Lundström , P. Halvarsson , P. Erixon , and A. Collado . 2010. “A Sensitive and Reliable Restriction Enzyme Assay to Distinguish Between the Mosquitoes *Culex torrentium* and *Culex pipiens* .” Medical and Veterinary Entomology 24: 142–149. 10.1111/j.1365-2915.2010.00871.x. 20444079

[ece374061-bib-0030] Hothorn, T. , F. Bretz , and P. Westfall . 2008. “Simultaneous Inference in General Parametric Models.” Biometrical Journal 50: 346–363. 10.1002/bimj.200810425. 18481363

[ece374061-bib-0031] Juliano, S. A. 2009. “Species Interactions Among Larval Mosquitoes: Context Dependence Across Habitat Gradients.” Annual Review of Entomology 54: 37–56. 10.1146/annurev.ento.54.110807.090611. PMC2664081 19067629

[ece374061-bib-0032] Kitching, R. L. 2000. Food Webs and Container Habitats: The Natural History and Ecology of Phytotelmata. Cambridge University Press.

[ece374061-bib-0033] Koenraadt, C. J. M. , and L. C. Harrington . 2008. “Flushing Effect of Rain on Container‐Inhabiting Mosquitoes *Aedes aegypti* and *Culex pipiens* (Diptera: Culicidae).” Journal of Medical Entomology 45: 28–35. 10.1093/jmedent/45.1.28. 18283939

[ece374061-bib-0034] Krause, J. , and G. D. Ruxton . 2002. Living in Groups. Oxford University Press.

[ece374061-bib-0035] LaDeau, S. L. , B. F. Allan , P. T. Leisnham , and M. Z. Levy . 2015. “The Ecological Foundations of Transmission Potential and Vector‐Borne Disease in Urban Landscapes.” Functional Ecology 29: 889–901. 10.1111/1365-2435.12487. 26549921 PMC4631442

[ece374061-bib-0036] Lawler, S. P. 2017. “Environmental Safety Review of Methoprene and Bacterially‐Derived Pesticides Commonly Used for Sustained Mosquito Control.” Ecotoxicology and Environmental Safety 139: 335–343. 10.1016/j.ecoenv.2016.12.038. 28187397

[ece374061-bib-0037] Lenth, R. 2022. “Emmeans: Estimated Marginal Means, Aka Least‐Squares Means. R Package Version 1.9.0.” https://cran.r‐project.org/package=emmeans.

[ece374061-bib-0038] Lindstedt, C. , L. Murphy , and J. Mappes . 2019. “Antipredator Strategies of Pupae: How to Avoid Predation in an Immobile Life Stage?” Philosophical Transactions of the Royal Society, B: Biological Sciences 374: 20190069. 10.1098/rstb.2019.0069. PMC6711284 31438812

[ece374061-bib-0039] Liu, X. , Baimaciwang , Y. Yue , et al. 2019. “Breeding Site Characteristics and Associated Factors of *Culex pipiens* Complex in Lhasa, Tibet, P. R. China.” International Journal of Environmental Research and Public Health 16: 1407. 10.3390/ijerph16081407. 31003560 PMC6517927

[ece374061-bib-0040] Lucas, E. A. , and W. S. Romoser . 2001. “The Energetic Costs of Diving in *Aedes aegypti* and *Aedes albopictus* Pupae.” Journal of the American Mosquito Control Association 17: 56–60. 11345420

[ece374061-bib-0041] Lüdecke, D. , M. S. Ben‐Shachar , I. Patil , P. Waggoner , and D. Makowski . 2021. “Performance: An R Package for Assessment, Comparison and Testing of Statistical Models.” Journal of Open Source Software 6: 3139. 10.21105/joss.03139.

[ece374061-bib-0042] Lutz, E. K. , K. T. Ha , and J. A. Riffell . 2020. “Distinct Navigation Behaviors in *Aedes*, *Anopheles* and *Culex* Mosquito Larvae.” Journal of Experimental Biology 223: jeb221218. 10.1242/jeb.221218. 32127378 PMC7132834

[ece374061-bib-0043] Medlock, J. M. , and K. R. Snow . 2008. “Natural Predators and Parasites of British Mosquitoes – A Review.” Journal of the European Mosquito Control Association 25: 1–11.

[ece374061-bib-0044] Montarsi, F. , S. Martini , M. Dal Pont , et al. 2013. “Distribution and Habitat Characterization of the Recently Introduced Invasive Mosquito *Aedes koreicus* [*Hulecoeteomyia koreica*], a New Potential Vector and Pest in North‐Eastern Italy.” Parasites & Vectors 6: 292. 10.1186/1756-3305-6-292. 24457085 PMC3852218

[ece374061-bib-0045] Moorefield, H. H. 1951. “Sexual Dimorphism in Mosquito Pupae.” Mosquito News 11: 175–177.

[ece374061-bib-0046] Nayar, J. K. , and A. Ali . 2003. “A Review of Monomolecular Surface Films as Larvicides and Pupicides of Mosquitoes.” Journal of Vector Ecology 28: 190–199. 14714668

[ece374061-bib-0047] Oksanen, J. , G. Simpson , F. Blanchet , et al. 2025. “Vegan: Community Ecology Package. R Package Version 2.7–2.” https://cran.r‐project.org/package=vegan.

[ece374061-bib-0048] R Core Team . 2025. R: A Language and Environment for Statistical Computing. R Foundation for Statistical Computing. https://www.r‐project.org/.

[ece374061-bib-0049] Ratnayake, O. C. , N. Chotiwan , K. Saavedra‐Rodriguez , and R. Perera . 2023. “The Buzz in the Field: The Interaction Between Viruses, Mosquitoes, and Metabolism.” Frontiers in Cellular and Infection Microbiology 13: 1128577. 10.3389/fcimb.2023.1128577. 37360524 PMC10289420

[ece374061-bib-0050] Rodríguez‐Prieto, I. , E. Fernández‐Juricic , and J. Martín . 2006. “Anti‐Predator Behavioral Responses of Mosquito Pupae to Aerial Predation Risk.” Journal of Insect Behavior 19: 373–381. 10.1007/s10905-006-9033-4.

[ece374061-bib-0051] Rolff, J. , P. R. Johnston , and S. Reynolds . 2019. “Complete Metamorphosis of Insects.” Philosophical Transactions of the Royal Society, B: Biological Sciences 374: 20190063. 10.1098/rstb.2019.0063. PMC6711294 31438816

[ece374061-bib-0052] Romero‐Ferrero, F. , M. G. Bergomi , R. C. Hinz , F. J. H. Heras , and G. G. de Polavieja . 2019. “Idtracker.Ai: Tracking All Individuals in Small or Large Collectives of Unmarked Animals.” Nature Methods 16: 179–182. 10.1038/s41592-018-0295-5. 30643215

[ece374061-bib-0053] Romoser, W. S. 1975. “Buoyancy and Ventilation in *Aedes aegypti* (L.) Pupae (Diptera: Culicidae).” Journal of Medical Entomology 12: 547–550. 10.1093/jmedent/12.5.547. 1223305

[ece374061-bib-0054] Saha, N. , G. Aditya , S. Banerjee , and G. K. Saha . 2012. “Predation Potential of Odonates on Mosquito Larvae: Implications for Biological Control.” Biological Control 63: 1–8. 10.1016/j.biocontrol.2012.05.004.

[ece374061-bib-0055] Service, M. W. 1968. “The Ecology of the Immature Stages of *Aedes detritus* (Diptera: Culicidae).” Journal of Applied Ecology 5: 613–630. 10.2307/2401636.

[ece374061-bib-0056] Shaalan, E. A.‐S. , and D. V. Canyon . 2009. “Aquatic Insect Predators and Mosquito Control.” Tropical Biomedicine 26: 223–261. 20237438

[ece374061-bib-0057] Shuey, J. A. , A. J. Bucci , and W. S. Romoser . 1987. “A Behavioral Mechanism for Resting Site Selection by Pupae in Three Mosquito Species.” Journal of the American Mosquito Control Association 3: 65–69. 3504897

[ece374061-bib-0058] Simon, C. , F. Frati , A. Beckenbach , B. Crespi , H. Liu , and P. Flook . 1994. “Evolution, Weighting, and Phylogenetic Utility of Mitochondrial Gene Sequences and a Compilation of Conserved Polymerase Chain Reaction Primers.” Annals of the Entomological Society of America 87: 651–701. 10.1093/aesa/87.6.651.

[ece374061-bib-0060] Tandina, F. , O. Doumbo , A. S. Yaro , S. F. Traoré , P. Parola , and V. Robert . 2018. “Mosquitoes (Diptera: Culicidae) and Mosquito‐Borne Diseases in Mali, West Africa.” Parasites & Vectors 11: 467. 10.1186/s13071-018-3045-8. 30103823 PMC6090629

[ece374061-bib-0061] Taylor, R. , L. A. Messenger , T. A. Abeku , S. E. Clarke , R. S. Yadav , and J. Lines . 2024. “Invasive *Anopheles stephensi* in Africa: Insights From Asia.” Trends in Parasitology 40: 731–743. 10.1016/j.pt.2024.06.008. 39054167

[ece374061-bib-0062] Timmermann, S. E. , and H. Briegel . 1999. “Larval Growth and Biosynthesis of Reserves in Mosquitoes.” Journal of Insect Physiology 45: 461–470. 10.1016/S0022-1910(98)00147-4. 12770329

[ece374061-bib-0063] Torrents, J. , T. Costa , and G. G. de Polavieja . 2026. “New Idtracker.Ai: Rethinking Multi‐Animal Tracking as a Representation Learning Problem to Increase Accuracy and Reduce Tracking Times.” eLife 14: RP107602. 10.7554/eLife.107602.4. 41983457 PMC13082788

[ece374061-bib-0064] Truman, J. W. 2019. “The Evolution of Insect Metamorphosis.” Current Biology 29: R1252–R1268. 10.1016/j.cub.2019.10.009. 31794762

[ece374061-bib-0065] Venables, W. N. , and B. D. Ripley . 2002. Modern Applied Statistics With S. Springer Science & Business Media.

[ece374061-bib-0066] Vezzani, D. 2007. “Review: Artificial Container‐Breeding Mosquitoes and Cemeteries: A Perfect Match.” Tropical Medicine & International Health 12: 299–313. 10.1111/j.1365-3156.2006.01781.x. 17300639

[ece374061-bib-0067] Vijayakumar, K. , T. K. Sudheesh Kumar , Z. T. Nujum , F. Umarul , and A. Kuriakose . 2014. “A Study on Container Breeding Mosquitoes With Special Reference to *Aedes* (*Stegomyia*) *Aegypti* and *Aedes albopictus* in Thiruvananthapuram District, India.” Journal of Vector Borne Diseases 51: 27–32. 24717199

[ece374061-bib-0068] Washburn, J. 1995. “Regulatory Factors Affecting Larval Mosquito Populations in Container and Pool Habitats: Implications for Biological Control.” Journal of the American Mosquito Control Association 11: 279–283. 7595462

[ece374061-bib-0069] Weaver, S. C. , C. Charlier , N. Vasilakis , and M. Lecuit . 2018. “Zika, Chikungunya, and Other Emerging Vector‐Borne Viral Diseases.” Annual Review of Medicine 69: 395–408. 10.1146/annurev-med-050715-105122. PMC6343128 28846489

[ece374061-bib-0070] Wei, Y.‐L. , Z. Wu , R.‐L. Li , and F. Tang . 2026. “Review of Selected Mosquito‐Borne Diseases: Arboviruses (Dengue, Chikungunya, Zika, West Nile, Japanese Encephalitis, Yellow Fever) and Parasitic Diseases (Malaria, Lymphatic Filariasis).” Frontiers in Public Health 13: 1712094. 10.3389/fpubh.2025.1712094. 41648764 PMC12868167

[ece374061-bib-0071] Wickham, H. 2016. ggplot2: Elegant Graphics for Data Analysis. Springer.

[ece374061-bib-0072] Wilkerson, R. C. , Y.‐M. Linton , and D. Strickman . 2021. Mosquitoes of the World. Johns Hopkins University Press.

[ece374061-bib-0073] Wilson, A. L. , O. Courtenay , L. A. Kelly‐Hope , et al. 2020. “The Importance of Vector Control for the Control and Elimination of Vector‐Borne Diseases.” PLoS Neglected Tropical Diseases 14: e0007831. 10.1371/journal.pntd.0007831. 31945061 PMC6964823

